# Reducing Boolean networks with backward equivalence

**DOI:** 10.1186/s12859-023-05326-9

**Published:** 2023-05-23

**Authors:** Georgios A. Argyris, Alberto Lluch Lafuente, Mirco Tribastone, Max Tschaikowski, Andrea Vandin

**Affiliations:** 1 Department of Applied Mathematics and Computer Science, Technical University of Denmark, Lyngby, Denmark; 2 SysMA Unit, IMT School for Advanced Studies, Lucca, Italy; 3 Department of Computer Science, University of Aalborg, Aalborg, Denmark; 4 Department of Excellence EMbeDS and Institute of Economics, Sant’Anna School for Advanced Studies, Pisa, Italy

**Keywords:** Boolean network, Model reduction, State-space generation, Attractors analysis, Partition refinement

## Abstract

**Background:**

Boolean Networks (BNs) are a popular dynamical model in biology where the state of each component is represented by a variable taking binary values that express, for instance, activation/deactivation or high/low concentrations. Unfortunately, these models suffer from the state space explosion, i.e., there are exponentially many states in the number of BN variables, which hampers their analysis.

**Results:**

We present Boolean Backward Equivalence (BBE), a novel reduction technique for BNs which collapses system variables that, if initialized with same value, maintain matching values in all states. A large-scale validation on 86 models from two online model repositories reveals that BBE is effective, since it is able to reduce more than 90% of the models. Furthermore, on such models we also show that BBE brings notable analysis speed-ups, both in terms of state space generation and steady-state analysis. In several cases, BBE allowed the analysis of models that were originally intractable due to the complexity. On two selected case studies, we show how one can tune the reduction power of BBE using model-specific information to preserve all dynamics of interest, and selectively exclude behavior that does not have biological relevance.

**Conclusions:**

BBE complements existing reduction methods, preserving properties that other reduction methods fail to reproduce, and vice versa. BBE drops all and only the dynamics, including attractors, originating from states where BBE-equivalent variables have been initialized with different activation values The remaining part of the dynamics is preserved exactly, including the length of the preserved attractors, and their reachability from given initial conditions, without adding any spurious behaviours. Given that BBE is a model-to-model reduction technique, it can be combined with further reduction methods for BNs.

**Supplementary Information:**

The online version contains supplementary material available at 10.1186/s12859-023-05326-9.

## Background

Boolean networks (BNs) are a popular model in systems biology where the dynamics is qualitatively associated with two levels. These may express, for instance, on/off behavior in gene regulation or high/low concentrations of molecular compounds [1]. In a BN, the state is defined as a vector of Boolean variables, each representing a distinct component of the system under consideration. The time evolution of each variable is governed by an update function, i.e., a Boolean expression that encodes how the other variables affect the change of state at each (discrete) time step [2–5]. In the main text we focus on synchronous BNs whereby the next state is obtained by applying all the update functions to the activation values of the current state. However, in the supplementary material we show how our approach can be applied also to BNs with partially asynchronous update schema (see, e.g., the *priority classes* supported by the popular tool GINsim [6] as described in [7]).

From a computational viewpoint, BNs are challenging to analyze. For example, the state space of the network, known as the state transition graph (STG), has exponential size in the number of variables. Thus, a full enumeration of the state space is possible only for networks of limited size. Another relevant type of analysis concerns the computation of attractors, i.e., those sets of states toward which the system tends to evolve and remain [8, 9]; these are often associated with biologically intelligible conditions of the system under study such as cell differentiation [3, 10]. Attractor identification is NP-hard [11] and, even if efficient tools have been developed [12], they do not scale well for large BNs.

These computational difficulties have motivated the development of reduction methods to ease BN analysis. Available techniques can be classified in three families according to the type of reduction: (i) by reasoning directly on the BN structure [4, 13–16]; (ii) by reducing the underlying STG [5, 17]; (iii) by transforming a BN into other formalisms for which specific reduction techniques are available [18, 19]. The latter two classes suffer two main limitations. First, STG-based reductions are still subject to state space explosion since they require the full enumeration of the state space to start with. Second, reductions via other formalisms may not be complete in the sense that the dynamics of the original BN and of the transformed model are not equivalent, hence some reductions may be missed (see Additional file 1).

In the case of reduction methods at the BN level, popular examples are based on the notion of *variable absorption*, proposed originally in [14, 15]. The main idea is that certain BN variables can get removed by replacing their occurrences with their update functions. This is based on the assumption that those variables evolve over time scales that justify that they can be updated first in the model. Other methods remove *output/leaf* variables [4, 13] (variables that do not appear in the update functions of other variables) or *frozen* ones (variables that stabilize to the same value after some iterations independently of the initial conditions) [16].

**Fig. 1:**
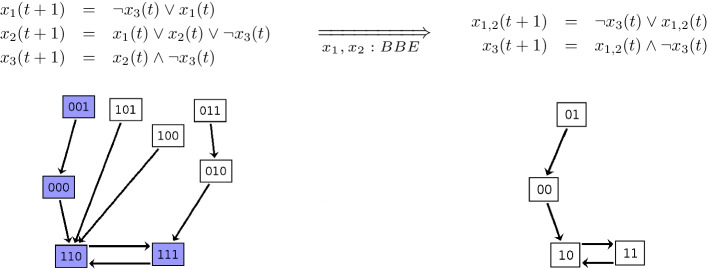
Boolean backward equivalence shown on a simple example. (Top-left) BN with three variables denoted by $x_1$, $x_2$, and $x_3$. (Bottom-left) The underlying STG. Each node is labelled by a vector that defines the state of each variable; a directed edge denotes a transition from a source state to a target state by a synchronous application of the update functions. States 110 and 111 form an attractor. (Top-right) Variables $x_1$ and $x_2$ can be shown to be BBE-equivalent by inspecting their update functions. If they have the same value in a state, i.e. $x_1(t)=x_2(t)$, then they will be equivalent for all successor states since $x_2(t+1) = x_1(t) \vee x_2(t) \vee \lnot x_3(t) =x_1(t) \vee x_1(t) \vee \lnot x_3(t)=x_1(t) \vee \lnot x_3(t)=x_1(t+1)$. Based on this, a reduced BN can be obtained by considering a representative variable for each block and rewriting the corresponding update functions in terms of those representatives (here the representative variable is denoted by $x_{1,2}$). (Bottom-right) The underlying STG agrees with the original one on all states that have equal values for variables in the same block (purple nodes in bottom-left panel). Instead, any other state (i.e. those where variables in the same BBE block have different value), is removed. The criteria for BBE only involve checks for the update functions of the original model, such that the generation of original STG can be circumvented

Here we present a complementary type of reduction method based on the computation of a partition of the variables in the BN, whereby the future dynamics of variables in a block of the partition are equal whenever they start from the same condition. This can be convenient, for instance, if one is interested in studying the dynamics due to simultaneous activation or deactivation of groups of variables [20] (see also the case studies presented in the *Results and discussion* section). We call this kind of relation a *Boolean backward equivalence* (BBE) because it is defined analogously to the notion of backward bisimulation for Markov chains [21], more recently extended to chemical reaction networks [19, 22] and ordinary differential equations [23]. Recently, it has been shown how this backward notion can be given also for linear differential algebraic equations (DAE) [24, 25]. Using DAE terminology, such backward notion has been related to the preservation of invariant subspaces.

Every reduction technique comes with its own intuitive interpretation. For example, if we consider variable absorption mentioned above, it is intuitively based on the idea of fast/slow decomposition: it is biologically plausible to absorb a variable in the update function of another when the former fires faster than the latter. BBE is based on the following three orthogonal considerations:

- BBE allows the modeler to discover chains of variables that, under some initialization conditions, describe the same dynamics. This might be interesting, e.g., to estimate the quality of a model: large BBE reductions might signal excessive redundancy in the model.
- As mentioned above, the modeler might be interested only in dynamics where two or more variables have simultaneous (de)activation value (see, e.g., [20]). The T-LGL case study in section *Results and discussion* further discusses this.
- In [23], it has been shown that this backward notion corresponds to Cardelli’s *emulation* [26] which enables to relate a complex model with a simpler one. Interestingly, [26] discusses how emulation can be given an evolutionary interpretation. In fact, an original model can express all the dynamics of the reduced model. In addition, the original model can also express all additional dynamics coming from states where variables related by emulation have different activation values (not permitted in the reduced model because variables related by emulation get collapsed in the same reduced one). Given this richer dynamics of the original model, Cardelli uses selected case studies in [26] to argue how the original model can be seen as an evolved version of the reduced one. We do not further investigate this aspect for BBE. However, given that BBE is based as well on the mentioned backward notions, it is not surprising that there exists a similar relation among the dynamics expressed by the original and reduced model (cf Fig. 1).

The criteria for a candidate partition of variables to be a BBE are encoded into a *satisfiability* problem over the expressions of the BN’s update functions: we synthesise a Boolean expression involving BN variables and check whether there exists at least one combination of truth values for the variables that makes such expression true. This type of test can be effectively implemented using tools known as SAT solvers [27].

If a partition is a BBE, a reduced BN can be obtained by choosing and maintaining only a representative variable for each partition block, and renaming all variables in the remaining update functions with the representative one from their block. The STG of the reduced network exactly preserves the original dynamics for all states that have equal values across variables in the same block (Fig. 1). Importantly, however, the reduction method does not require the generation of the original STG, making it possible to obtain a reduced STG also from instances that would not be analyzable due to their massive size.

A crucial property satisfied by BBE is that there exists a *maximal* reduction for each BN, i.e., the coarsest BBE partition. This can be computed using a partition-refinement algorithm in a similar fashion as in Markov chains [28], reaction networks [22] and differential equations [23]. The algorithm essentially builds upon a fundamental result in computer science to prove equivalences in formal languages [29]. Given an initial partition of variables, the algorithm splits the blocks of the partition to compute its coarsest refinement that satisfies the BBE criteria. Thus, the maximal reduction is obtained when all variables are in the same unique block of the initial partition. However, the possibility of arbitrarily choosing the initial partition unlocks model-specific reduction queries that preserve the dynamics of user-defined variables. For example, in typical BN models of signaling pathways [30, 31], certain variables may represent the input signals to upstream components such as receptors. Formally, inputs may be detected because their update functions are constants that represent the values of such inputs. In this case, a possibly more biologically relevant initial partition may separate inputs from the other variables, obtaining *input-separated* (IS) reductions.

Our partition-refinement algorithm takes a polynomial number of steps as a function of the number of BN variables. At each iteration, it queries a SAT solver to check for the BBE criteria. If the query is satisfiable, i.e., the current partition is not a BBE, the returned assignment is used to split the current partition and perform another iteration; if the query is unsatisfiable, it returns the current partition as the coarsest BBE refinement of the initial one. We fully develop our approach in the *Methods* section and in Additional file 2.

Interestingly, although our algorithm is theoretically as complex as SAT solving, it behaves effectively in practice. Using a prototype implementation available within the software tool ERODE [32], we demonstrate its performance on a large-scale validation across 86 BN models from two well-known repositories [6, 33]. We show that almost all BNs can be reduced by BBE, providing speed-ups for the computation of STGs and attractors by more than three orders of magnitude. In some cases, BBE could render the analysis feasible in instances that originally issued out-of-memory errors or that were stopped after long time outs. This comes at the cost that part of the original dynamics is lost. In particular, in the STG we preserve all and only the states where variables within the same BBE-block have the same value, and transitions among them. From this, and from the properties of BBE, we also get that the method preserves all and only the attractors containing at least one preserved state. This confirms that BBE is complementary to existing reduction techniques for BNs. Indeed, in several areas of science and engineering, it is common to have reduction techniques that:

- Preserve all dynamics but might add spurious ones. An example is [14] which preserves all attractors but might create new spurious ones. These often come with the name of *over-approximations* (this is because, e.g., [14] might over-approximate the set of attractors of a model by computing a larger set containing all original ones, plus some spurious ones;
- Do not preserve all the dynamics, but guarantee to not add spurious ones, like BBE. These often come with the name of *under-approximations* (this is because, e.g., BBE might under-approximate the set of attractors of a model by computing a smaller set containing only original ones, but potentially not all).

These two families of techniques are not comparable. They might be jointly used to obtain upper-bounds (the case of [14]), and lower-bounds (the case of BBE) on the actual number of attractors in a model.

This paper extends the previous conference version [34]. All numerical experiments have been redesigned by adding an additional model repository, by performing a large-scale validation of the analysis speed-ups offered by BBE, and by considering a more recent and efficient tool for identification of attractors. We have also performed a new large-scale validation on randomly generated BNs. Finally, we have generalised the theory to support also BNs with partially asynchronous update schema, and we have added in Additional file 3 a new case study considering one such BN.

## Results and discussion

In this section, we perform a large-scale validation of BBE. We first check its reduction power on published models from the literature, and then we demonstrate how it facilitates the analysis tasks of STG generation and attractor computation. In particular, we show how BBE brings important analysis speed-ups, both in terms of STG generation, and attractor analysis. In several cases, BBE enables the analysis of models that were originally intractable due to their complexity.

After this, we use two selected case studies to show how one can tune the reduction power of BBE to preserve or exclude specific dynamics of interest.

*Toolchain.* We implemented our method in ERODE [32], a freely available software for the modeling, analysis, and reduction of biological systems modelled in terms of BNs [34], differential equations [35], and chemical reaction networks [19]. ERODE integrates the SAT solver Z3 [36]. Thanks to importing/exporting functionalities, we let ERODE interact with the COLOMOTO Notebook [37, 38], which integrates several tools for modeling and analysis of BNs. STG generation is performed using the tool PyBoolNet [39], while attractor identification is performed using the SAT-based tool BNS [12].^1^

*Configuration.* All experiments were conducted on a machine equipped with an Intel Xeon(R) 2.80 GHz processor and 32 GB RAM. We imposed an arbitrary timeout of 8 h for each task, after which we terminated the analysis. We refer to these cases as *time-out*, while we use *out-of-memory* if the execution issued a memory error.

We conducted our investigation using two model repositories: GINsim repository [6] (http://ginsim.org/models_repository), which contains 83 models, and the Biomodels repository [42] (https://www.ebi.ac.uk/biomodels/), which contains 24 models, obtaining overall 98 distinct models (9 appeared in both repositories). From these, we restricted only to models with input variables, obtaining 86 models. In other words, we considered about 92% of the models available in the two respositories. This selection was done to avoid favouring BBE: in BNs without inputs, IS initial partitions correspond to the maximal ones, which, as the name says, allow for the best possible BBE reduction of a model in terms of aggregation power. Part of these 86 models, 45, are multi-valued networks, i.e. logical models wherein some variables take more than two activation statuses, e.g., $\{0,1,2\}$ for *low*, *medium*, or *high* concentration respectively (see, e.g., [43]). We transform such models in dynamically equivalent BNs by applying a so-called *booleanization* technique [44], supported by GINsim [40].

We consider two reduction scenarios relevant to input variables, using *maximal* and *input-separated* (IS) initial partitions like $\mathcal {H}_0$ in Eq. (1) and $\mathcal {H}_0'$ in Eq. (2), respectively. In Additional file 4 we perform a similar analysis on randomly generated BNs.

### Large-scale validation

**Fig. 2:**
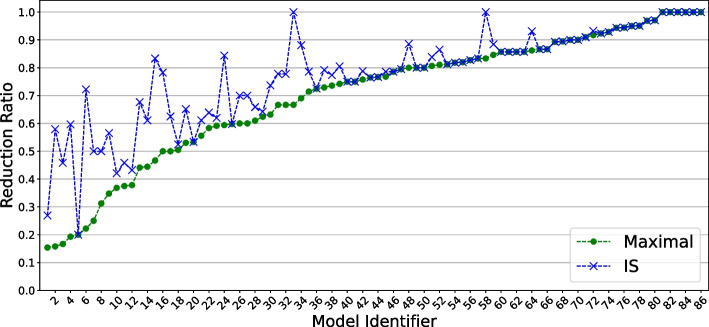
Large-scale validation: reduction power. The x-axis provides model identifiers for the 86 considered models (only even ones are shown due to space constraints), while the y-axis refers to reduction ratios “reduced variables over original ones”. The green dots denote the reduction ratios, in increasing order, for maximal reductions. Using the same ordering, the blue crosses denote the reduction ratios for IS reductions. Only 6 models do not admit any BBE reduction, while two more models (33 and 58) do not admit IS reduction

*Large-scale validation: reduction power.* We begin by addressing the reduction power of BBE. For this, we consider the *reduction ratios* (variables in the reduced BN over the variables in the original one) obtained on all models.

Figure 2 displays the reduction ratios for both the maximal and IS reductions. We observe that almost all models can be reduced by BBE, in particular 93% admit a maximal reduction, while 91% admit an IS one. The reduction ratios distribute almost uniformly from 0.15 to 1.00 (no reduction), with average reduction ratio of 0.70 and 0.77 for maximal and IS reductions, respectively. For most models, the maximal and IS reduction ratios do not change significantly, meaning that BBE is effective also when we prevent input variables from merging with internal variables. All detailed results of this analysis can be found in Additional file 5: Table S3.

**Fig. 3:**
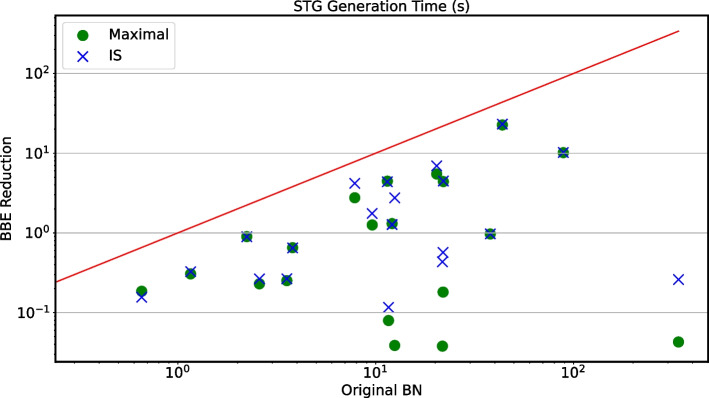
Large-scale validation: STG generation speed-up. Comparison of STG generation between BBE reductions and original BNs. We omit models with 10 or fewer variables, where the runtimes are not particularly informative because STG generation is trivial. Furthermore, we omit models with more than 60 variables, where STG generation fails with *out-of-memory* for both the original models and their reductions. Overall, we obtain 33 models, from which here we focus only on the 20 ones for which the STG generation succeeded in both the original and reduced models, while Fig. 4 focuses on the remaining 13. The *x*-axis refers to the generation time for original models, while the *y*-axis refers to that for reduced models, using green circles and blue crosses for maximal and IS reductions, respectively. The runtimes are averaged over 3 runs

**Fig. 4:**
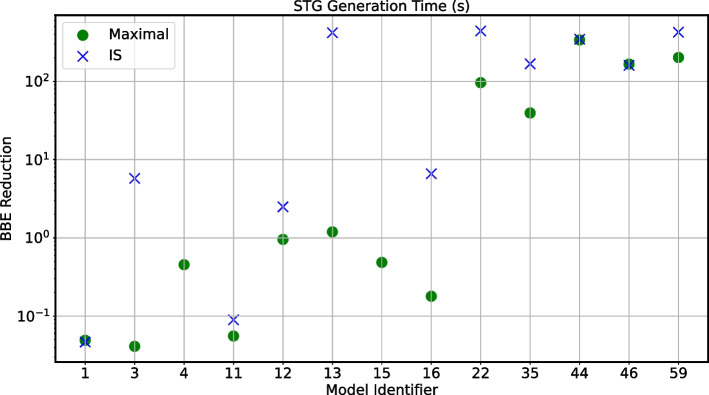
Large-scale validation: STG generation speed-up. STG generation time for the maximal and the IS reductions. We consider the 13 models omitted in Fig. 3 because the STG generation failed for the original models. The x-axis refers to the model identifier from Fig. 2, while the y-axis refers to the generation time for the reduced models, using green circles and blue crosses for maximal and IS reductions, respectively. The runtimes are averaged over 3 runs

*Large-scale validation: STG generation speed-up.* We hereby demonstrate the speed-ups that BBE provides to STG generation on a selection of the considered models. Figure 3 focuses on the 20 models with more than 10 variables for which the STG generation succeeded in both the original and reduced models, while Fig. 4 focuses on the 13 ones where the STG generation failed on the original model and succeeded in the maximal or IS reduction. On the used machine, STG generation failed for models with 24 or more variables. Therefore, Fig. 3 focuses on models with less than 24 variables, while Fig. 4 focuses on models with 24 or more variables for which at least one reduction had less than 24 variables.

The red line in Fig. 3 marks the area where the reduction would not bring speed-ups. We can see that all points are below the line, with instances showing more than two orders of magnitude difference between the original and reduced runtimes. This proves that BBE can effectively lead to faster STG generation. All cases where the dots and crosses overlap refer to models where the two reductions coincide.

We now consider the 13 models in Fig. 4 where STG generation was not feasible for the original models. We note that the generation succeeded for all maximal reductions, while it failed for two IS ones. As denoted by the model identifiers in the x-axis, these are models 4 and 15 in Fig. 2, where the IS reductions have 34 and 25 variables, respectively. The largest runtime is 441 s for the IS reductions, and 338 s for the maximal ones. Detailed results are presented in Additional file 5: Table S4.

**Fig. 5:**
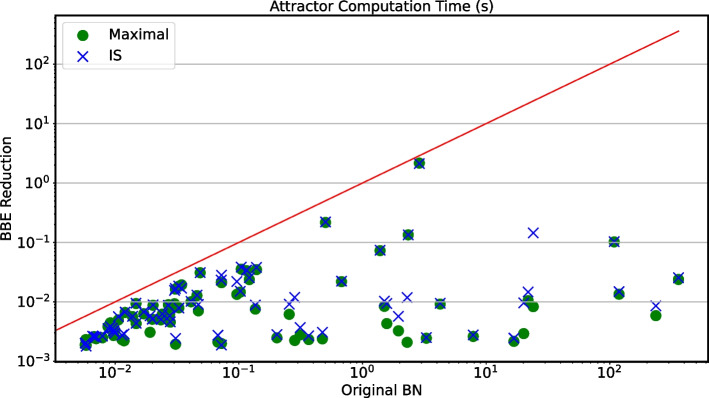
Large-scale validation: Attractor computation speed-up. Attractor computation time of the original models versus the one of maximal and IS reductions. Out of the 86 models from Fig. 2 we select the 78 admitting both maximal and IS reduction. The figure further omits models 18 and 29 from Fig. 2 for which the analysis failed for the original model due to *time-out*. The x-axis refers to the analysis time for original models, while the y-axis refers to that for reduced models, using green circles and blue crosses for maximal and IS reductions, respectively. The runtimes are averaged over 3 runs

*Large-scale validation: Attractor computation speed-up* Fig. 5 studies the speed-ups that BBE provided to the computation of attractors on the models from Fig. 2. The plot has the same structure as Fig. 3. We observe that, in several cases, we have significant analysis speed-ups. In particular, we note how the dots and crosses spread to the right, due to original runtimes in the order of $10^3\,\text {s}$, while they hardly go up beyond $1\,\text {s}$ for runtimes on reduced models. Furthermore, models 18 and 29 from Fig. 2 are omitted here because the analysis failed on the original models. Instead, the analysis of their maximal reductions required at most 0.15 s, and that of their IS reductions at most 2.5 s. Detailed results are given in Additional file 5: Table S5.

*Large-scale validation: Interpretation.* BBE can successfully reduce a large amount of models. For the original models, the state space explosion prevents full state space exploration in many cases, and hampers the identification of the attractors. This is mitigated in practice by BBE, with extreme cases where BBE made analysis feasible whereas the original models were intractable. As shown in Additional file 5: Table S5, part of the attractors are lost in the reduced models, namely those not involving constant states on the computed BBE (see Methods section). Table S5 shows cases like model 18 or 29, whose attractors could not be computed at all without BBE reduction. At the same time, the table shows that, by using the *default* IS or maximal initial partitions, a large part of the attractors might be lost (because, according to our theory, involve STG states where variables belonging to the same BBE block have different value). In Additional file 4: Fig. S7 we provide this information graphically for the BNs from the two repositories, and for randomly generated ones. This can be mitigated by devising *refined* initial partitions for the model and problem at hand. This is exemplified and discussed in greater detail in the next section where we show how a modeler can easily devise refined initial partitions that may allow to preserve more attractors, or drop those that are not of interest.

### Case studies

In the previous part of this section we studied the aggregation power and the analysis speed-ups offered by BBE on 86 models from the literature. Here, instead, we use two selected case studies (MAPK, T-LGL) to show how one can tune, or *refine* the reduction power of BBE using model-specific information to preserve all dynamics of interest, and selectively exclude behavior that does not have biological relevance. Nevertheless, for completeness, we provide in Table 1 information on the analysis runtimes on all models (and their reductions) discussed in this section. We consider analysis runtimes on the models (*Original*), and on their IS (*IS*) and maximal (*Maximal*) reductions as done in the large-scale validation. Furthermore, we consider an additional reduction obtained using a refined initial partition discussed in the corresponding sections (*Refined*). STG generation failed on all models and reductions because they all have more than 24 variables. Indeed, we have previously discussed how, on the used machine, STG generation fails for models with 24 or more variables. Instead, attractor analysis succeeded on all models, with important speed-ups obtained for all reductions. For both models, the *IS* and *Maximal* cases have a particularly low analysis runtime. This is because, as we shall discuss next, several attractors are discarded in these reductions. Notably, despite the *Refined* reductions have speed-up factors of about two, as we shall see they preserve all attractors for MAPK, and all attractors *of interest* for T-LGL.

**Table 1:** Analysis runtimes (in seconds) for the models in section Case Studies

	MAPK				T-LGL			
	Original	IS	Maximal	*Refined*	Original	IS	Maximal	Refined
STG generation	Out-of-memory				Out-of-memory			
Atractor analysis	0.55	0.16	0.16	0.35	2.66	0.10	0.11	1.17

**Fig. 6:**
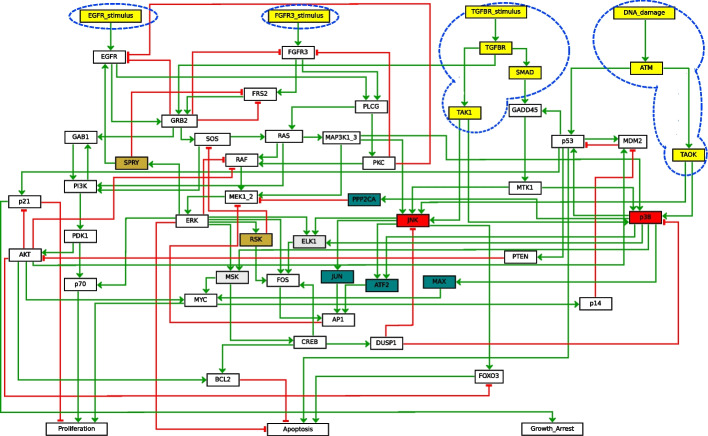
Graphical representation of the MAPK BN using GINsim. The background colors denote blocks of the maximal BBE (white background denotes singleton blocks). Instead, the blue dashed shapes denote blocks of the refined initial partition, vertical IS, where we omit the fifth large block containing all remaining nodes

*MAPK case study* We consider a BN model for Mitogen-Activated Protein Kinase (MAPK) from [45]. The model consists of tightly interconnected signalling pathways involved in diverse cellular processes, such as cell cycle, survival, apoptosis and differentiation. The BN is depicted in Fig. 6. It contains 53 variables, 4 of which being inputs ($EGFR\_stimulus$, $FGFR3\_stimulus$, $TGFBR\_stimulus$, and $DNA\_damage$), and has 40 attractors.

*MAPK: Maximal and IS reduction* The maximal BBE reduction of this model has 39 variables. The discovered blocks are visualized in Fig. 6 using different background colors. In particular, we note that the yellow block contains all inputs and five non-inputs variables, three related to $TGFBR\_stimulus$, and two related to $DNA\_damage$. Instead, the IS reduction has 41 variables, the only difference being that the block with inputs from the maximal reduction (Fig. 6) is split in three blocks: one for the inputs, one for the two non-input variables directly connected to the two right-most inputs, and one for the remaining non-input variables. In both cases, the reduced BNs have 17 attractors.

**Fig. 7:**
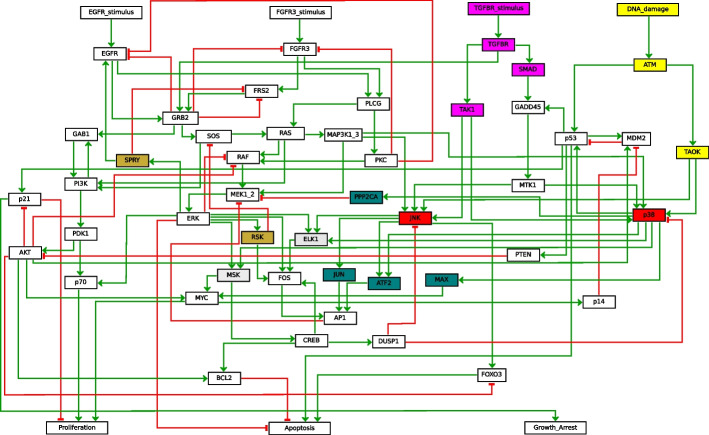
Graphical representation of the MAPK BN. Background colors denote blocks of the BBE obtained using the refined initial partition (white background denotes singleton blocks)

*MAPK: Refined reduction with vertical IS* We propose a third model-specific initial partition that considers inputs also *indirectly*. Intuitively, variables like $TGFBR$ depend only on the value assigned to an input ($TGFBR\_stimulus$). This reasoning can be iterated downward through the pathway, allowing to add also $TAK1$, and $SMAD$, until variables that depend on other (input) variables are met. In some sense, we can see $TGFBR$, $TAK1$, and $SMAD$ as indirect inputs. This is because, in a few iterations the value assigned to the corresponding input will be propagated to them, and they will not change value anymore. In other words, we use a block per input, each containing the input and all non-input variables only positively affected by the input or by variables in the block. That way, we obtain an initial partition denoted by the blue dashed shapes in Fig. 6, plus an additional fifth block containing all other variables. The rationale is that a variable only affected by an input will have the same truth value of the input, therefore it can be considered as a sort of indirect input. The obtained BBE is depicted in Fig. 7. The reduced BN contains 42 variables and preserves all 40 attractors.

**Fig. 8:**
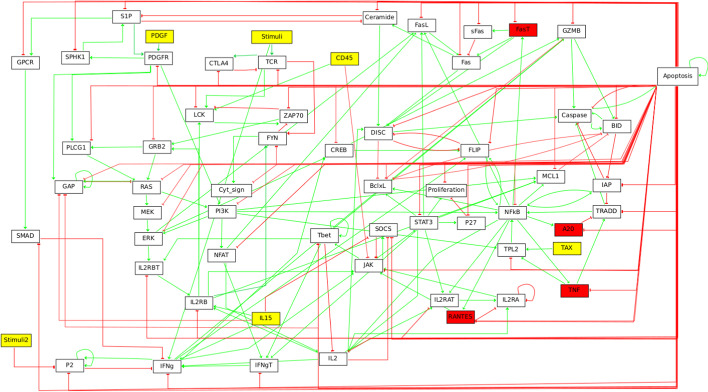
Graphical representation of the T-LGL BN using GINsim. Background colors denote blocks of both the maximal and IS BBE, which coincide (white background denotes singleton blocks)

*T-LGL case study* We consider a BN model for T-LGL from [20]. It refers to the disease T-LGL leukemia which features a clonal expansion of antigen-primed, competent, cytotoxic T lymphocytes (CTL). This BN is a signalling pathway, constructed empirically through extensive literature review, and determines the survival of CTL. The BN, depicted in Fig. 8, consists of 60 variables, 6 of which are inputs (the yellow nodes in Fig. 8). The model has 264 attractors.

*T-LGL: Maximal and IS reduction* The maximal and IS BBE coincide, as depicted in Fig. 8. We have only two non-singleton blocks: one consisting of all the inputs, and one consisting of $FasT$, $A20$, $TNF$, and $RANTES$. The reduced BN has 52 variables and 6 attractors, which means that most of the attractors are lost.

*T-LGL: Refined reduction* In [20], the authors discover that the simultaneous activation of the two input variables $IL15$ and $PDGF$ is sufficient to produce all dynamics of interest to them (namely, all the known so-called deregulations and signalling abnormalities).

In terms of initial partitions for BBE, we can encode the notion of *contemporary activation or deactivation* of the two inputs by using a model- and problem-specific initial partition where $IL15$ and $PDGF$ form a block. Furthermore, we assign every input to a singleton block, while all non-input variables belong to the same block, for a total of 56 blocks. It turns out that this initial partition is actually a BBE, which therefore does not get refined by our algorithm. The reduced BN has 120 attractors.

## Conclusion

Boolean backward equivalence (BBE) is an automatic reduction technique for Boolean networks (BNs) which exactly preserves dynamics of interest to the modeler by collapsing variables that are proven to have equal values in all states. The method, based on a partition refinement algorithm, can be tuned on a model- and problem-specific way by specifying which variables should be preserved using an appropriate choice of the initial partition. The approach is complementary to the state of the art. Roughly, in [4, 13], reduction is achieved by replacing variables with constants and propagating those in the transitions of somehow richer STGs or across the network, respectively. Thus, the reduced model cannot be used to investigate how changes in those variables affect the dynamics. In a BBE reduction, instead, variables are collapsed into blocks and the original dynamics is exactly recovered whenever variables in the same block are assigned equal values. These studies [4, 13] additionally remove the output [4] variables (also called leaf variables [13]). However, output variables sometimes are used to denote different “responses” by the modelled system [4, 30], therefore their removal might not always be appropriate.

In variable absorption [14, 15], the main assumption is that there are variables that are updated faster than others, therefore one class of variables can be assumed to be constant and absorbed if focusing on the dynamics of the other class. Unlike BBE, this can only increase the number of attractors. In particular, variable absorption preserves exactly all steady states (single-state attractors), while it might change the length of other attractors. Furthermore, new spurious attractors might be added. Instead, BBE might decrease the number of attractors (it discards all and only the attractors involving states where BBE-equivalent variables have different activation values), but all preserved attractors are preserved exactly, including their length and reachibility from (preserved) initial states, and no spurious ones are added. Regarding other relevant work, in [16], the authors identify variables that have the same value in attractors only, but, differently from BBE, might behave differently in other states of the STG.

We validated BBE on 86 BNs from two model repositories, providing reductions and analysis speed-ups in almost all cases. In some, BBE enabled the analysis of models which would be otherwise intractable. There were also instances for which the reduced model could not be analyzed. This calls for further research into more aggressive reductions; for example, in its current implementation multi-valued BNs are first translated into ordinary BNs, but this causes a blow-up in the number of variables. It is worth investigating approaches that circumvent the intermediate translation to reduce dimensionality. Another area of research concerns the different semantic interpretations of a BN. Currently, BBE supports BN with synchronous and partially asynchronous updates; we plan to investigate variants of BBE for probabilistic BNs.

## Methods

**Fig. 9:**
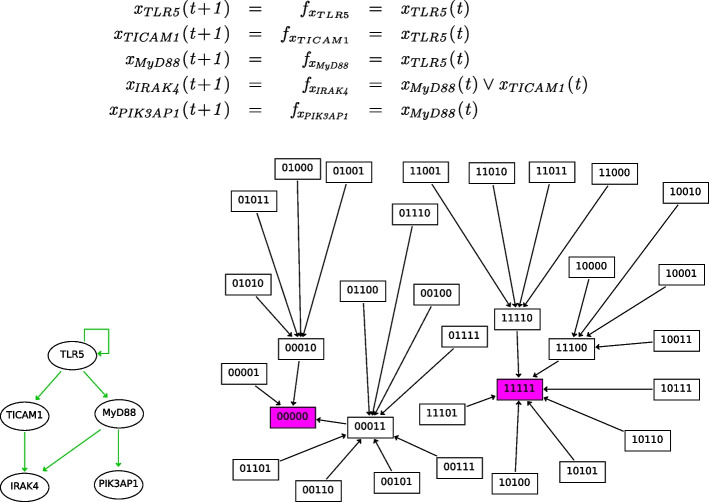
Excerpt of the BN from [30]. It refers to the receptor *TLR5* and its signalling to the four following genes: *TICAM1, MyD88, IRAK4, PIK3AP1*. When a virus infects an organism, the receptor *TLR5* receives the relevant antigen stimuli becoming active (the value of $x_{TLR5}$ turns from 0 to 1), and the signal is subsequently propagated to the other connected genes. (Top) The update functions of the BN. (Bottom-left) Variables are commonly depicted as nodes in a network while directed links represent influences between them. A directed link from a source variable to a target variable denotes that the source variable exists in the update function of the target variable. (Bottom-right) The corresponding STG, where we use purple to denote attractors

Here we explain the key steps of the reduction procedure on the BN in Fig. 9. Its customary graphical representation allows one to distinguish different kinds of variables depending on whether they appear in the update functions of other variables (as indicated by the green arrows). In the example, *TLR5* can be interpreted as an input because its state remains constant and unaffected by other variables. Inputs, which often denote external stimuli [4], are explicitly set by the modeler to perform experiment campaigns. Conversely, *IRAK4* and *PIK3AP1* can be considered output variables because they do not appear in the update functions of other variables.

*Step 1: initial partition.* Our reduction algorithm starts with the specification of an initial partition of variables. The idea behind initial partitions is that the modeler can force our algorithm to not collapse given variables, by placing them in different initial blocks. In the case studies presented in the *Results and discussion* section we see examples of user-specified initial partitions enabling analyses of interest on the considered models. This is how initial partitions shall be used, devising case-by-case useful ones. In order to favour a systematic large-scale validation of our approach, here we consider two examples of initial partitions whose computation can be easily automated: the *maximal* partition, where all variables are placed in the same block; and the *input-separated* (IS) one, where the inputs are separated from the other variables, i.e., we use an initial partition with two blocks, one for input variables and one for the other variables. ,^2^ In the example, these are respectively given by the partitions:

1 $$\begin{aligned} \mathcal {H}_0 = \big \{\{x_{ TLR5 }, x_{ TICAM1 }, x_{ MyD88 }, x_{ IRAK4 }, x_{ PIK3AP1 }\} \big \} \end{aligned}$$

and

2 $$\begin{aligned} \mathcal {H}'_0 = \big \{\{x_{ TLR5 }\}, \{x_{ TICAM1 }, x_{ MyD88 }, x_{ IRAK4 }, x_{ PIK3AP1 }\} \big \}. \end{aligned}$$

*Iterative step: splitting by the BBE condition.* At every iteration, the algorithm checks the BBE condition on the current partition. Formally, BBE is defined as a partition $X$ of variables that renders the following formula valid:

3 $$\begin{aligned} \Phi ^{\mathcal {H}} \equiv \left( \mathop {{\bigwedge }}\limits _{{\mathop {x,x' \in H_i}\limits ^{H_i \in \mathcal {H}}}} \Bigl ( x = x' \Bigr ) \right) \longrightarrow \mathop {{\bigwedge }}\limits _{{\mathop {x,x' \in H_i}\limits ^{H_i \in \mathcal {H}}}} \Bigl ( f_x = f_{x'} \Bigr ) \end{aligned}$$

This is a Boolean formula for: *whenever all variables in the same block have same value, they will not be distinguished in the next state*. In other words, $\Phi ^{\mathcal {H}}$ says that if for all partition blocks $H_i$ the variables in $H_i$ are equal, then the evaluations of update functions of variables in the same block stay equal. A SAT solver can determine if $\Phi ^{\mathcal {H}}$ is valid by checking the unsatisfiability of its negation. For example, given the $\mathcal {H}'_0$ partition in Eq. 2, one can obtain that $\lnot \Phi ^{\mathcal {H}'_0}$ is satisfiable (i.e., $\mathcal {H}'_0$ is not a BBE) because there exists the assignment *s* given by

$$\begin{aligned} s = ( s_{x_{ TLR5 }}, s_{x_{ TICAM1 }}, s_{x_{ MyD88 }}, s_{x_{ IRAK4 }}, s_{x_{ PIK3AP1 }}) = (1,0,0,0,0) \end{aligned}$$

for which, as it can be seen in the STG of Fig. 9, the next state $s'$ is

$$\begin{aligned} s' = ( s'_{x_{ TLR5 }}, s'_{x_{ TICAM1 }}, s'_{x_{ MyD88 }}, s'_{x_{ IRAK4 }}, s'_{x_{ PIK3AP1 }}) = (1,1,1,0,0). \end{aligned}$$

This assignment proves that variables $x_{ TICAM1 }$, $x_{ MyD88 }$, $x_{ IRAK4 }$, and $x_{ PIK3AP1 }$ cannot belong to the same block of a partition that satisfies the BBE criteria because despite having the same value (0) in the source state *s*, they differ in the target state $s'$. In addition, the assignment *s* suggests to split that block into two sub-blocks for which that assignment does not disprove the BBE condition: $x_{ TICAM1 }$ and $x_{ MyD88 }$ have same value in $s'$, as well as $x_{ IRAK4 }$ and $x_{ PIK3AP1 }$. Thus the algorithm will perform a new iteration with the refined partition

$$\begin{aligned} \mathcal {H}'_1 = \big \{ \{x_{ TLR5 }\}, \{x_{ TICAM1 }, x_{ MyD88 } \}, \{x_{ IRAK4 }, x_{ PIK3AP1 }\}\big \}. \end{aligned}$$

With this, $\lnot \Phi ^{\mathcal {H}'_1}$ is unsatisfiable, implying that $\mathcal {H}'_1$ is a BBE partition. In Theorem 2 from Additional file 2, we prove that this algorithm returns, for any initial partition, its unique coarsest refinement that satisfies the BBE condition (3). Overall, the algorithm takes at most *n* steps, where *n* is the number of BN variables; at every step, it iterates through the provided SAT assignment, if any is provided, to perform the splitting. Thus, overall the algorithm is as hard as SAT solving; however, the numerical evaluation presented in the *Results and discussion* section will show how it can effectively tackle BN models from the literature.

*BBE properties* As discussed in Fig. 1, given a BBE it is possible to construct a reduced BN where each variable represents a partition block (Proposition 4 from Additional file 2). The STG of the reduced BN agrees with the original STG on all, and only on, states that are *constant* on the partition, i.e., whose variables in the same block have the same value (Proposition 4 from Additional file 2). The reduction also preserves any attractor of the original BN which contains at least one state that is constant on the partition (Theorem 5 from Additional file 2). Thus, in particular the reduced BN maintains the exact length of the attractors that are preserved without introducing spurious dynamical behavior. Instead, all states non constant on the partition are dropped, as well as all attractors not containing any state constant on the BBE partition.

We use two examples to better explain the exact preservation of part of the attractors. Considering preserved attractors, we have seen in Fig. 1 that the two-states attractor of the original model (Fig. 1 bottom-left) is preserved in a two-states attractor in the reduced model (Fig. 1 bottom-right). This is the case for any preserved attractor; the number of states is preserved.

**Fig. 10:**
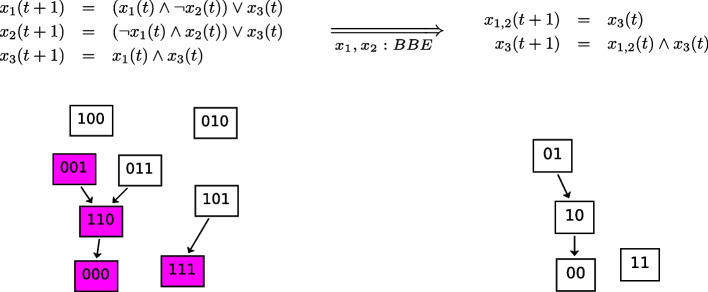
Boolean backward equivalence shown on a simple example: not all attractors are preserved. (Top-left) BN with three variables denoted by $x_1$, $x_2$, and $x_3$. (Bottom-left) The underlying STG. The model has 4 steady-state attractors (nodes 100, 010, 000, and 111). Two have same activation values for $x_1$ and $x_2$ (000, and 111), two have not. (Top-right) Variables $x_1$ and $x_2$ can be shown to be BBE-equivalent by inspecting their update functions. If they have the same value in a state, i.e. $x_1(t)=x_2(t)$, then they will be equivalent for all successor states. Based on this, a reduced BN can be obtained by considering a representative variable for each block and rewriting the corresponding update functions in terms of those representatives (here the representative variable is denoted by $x_{1,2}$). (Bottom-right) The underlying STG agrees with the original one on all states that have equal values for variables in the same block (purple nodes in bottom-left panel). Notably, the two attractors having same activation value for $x_1$ and $x_2$ are preserved, while the other two are dropped, as expected by our theory

As regards attractors that are not preserved, we provide in Fig. 10 (top-left) a simple BN with 3 variables ($x_1$, $x_2$, and $x_3$) and 4 attractors (steady-states, Fig. 10, bottom-left). Figure 10 (top-right) shows a BBE reduction of the model where $x_1$ and $x_2$ get collapsed. We can see in Fig. 10 (bottom-right) that 2 attractors are preserved in the BBE reduction, while the other 2 attractors belong to the part of the STG that is not preserved, and therefore are not present in the reduced BN. In particular, according to our theory, the two attractors where $x_1$ and $x_2$ are both 1 or both 0 are preserved. Instead, the other two attractors have different values for $x_1$ and $x_2$, and therefore are not preserved.

*Partially asynchronous BNs* In Additional file 2, we show how BBE can also be applied to partially asynchronous BNs. Here, we equip a BN with a partition $\mathcal {K}$ of its variables that we name *synchronization partition*. A new state is obtained by selecting one of the blocks *K* of $\mathcal {K}$, and then applying of the update functions of the variables in *K* only. The activation values of the other variables are not modified. Notably, this synchronization schema is supported, e.g., by popular BN analysis tools like GINsim [6] under the name of *priority classes* [7].^3^ In particular, BBE can be applied to such BNs with the caveat that the initial partition must be $\mathcal {K}$ or refinements of it. In Additional file 3 we apply BBE to a BN with partially asynchronous update schema.

## Supplementary Information

**Additional file 1**: Comparison with encoding-based reductions.

**Additional file 2**: Technical Results.

**Additional file 3**: Application of BBE to a BN with partially asynchronous schema.

**Additional file 4**: An application of BBE to randomly generated Boolean Networks.

**Additional file 5**: Tables with detailed results from large-scale experiments.

## Data Availability

Material to replicate the large-scale experiments, including all models, are available here: https://www.erode.eu/models/BMCBioInf_CMSB2021.zip

## Abbreviations

BN: Boolean network
BBE: Boolean backward equivalence
STG: State transition graph

## Footnotes

1 Among the tools available in the COLOMOTO notebook, we could have opted for GINsim [40] and BoolSim [41] for STG generation and attractor identification, respectively, for BNs with synchronous update schema. In both cases, we have opted for the tools with best performances according to preliminary experiments we conducted, not reported here.

2 We refer to [34] for a third example of initial partition, *input-distinguished* where inputs were further separated from each other. As exemplified in the case studies in the *Results and discussion* section, initial partitions should be defined by the modeler depending on the model at hand and on the properties to be studied. We discuss the maximal and IS partitions here to enable the large-scale validation of BBE discussed in the same section.

3 The dynamics of BNs considered in this paper are less general than those offered by GINsim using priority classes, as they also further allow to assign different priorities to the classes, and to update the variables within them asynchronously.
