# Supplementary material for: Reducing Boolean networks with backward equivalence
Source: BMC Bioinformatics. 2023 May 23;24(Suppl 1):212. doi: 10.1186/s12859-023-05326-9 (PMC10207686; doi:10.1186/s12859-023-05326-9)
Supplement: Supplementary file 1 — Additional file 1: Comparison with encoding-based reductions. [file 12859_2023_5326_MOESM1_ESM.pdf]

1 Comparison with encoding-based reductions

In this section we discuss how BN reduction techniques mediated by a translation into another formalism may miss certain reductions. In particular, we present a comparison with the approach from [19] based on ordinary differential equations (ODEs). Further details on such comparison can be found in [34]. The considered ODE-based approach first applies a so-called *odification* technique to encode a BN into an ODE system [47]; then it applies backward equivalence to ODEs, which is the ODE counterpart of BBE.

We consider the *TCR-TLR* model from [30], part of which adopted in the *Method* section. The equations for two of the variables in the model, *MyD88* and *IRAK4*, are given by:

$$x_{MyD88}(t+1) = x_{TLR5}(t)$$
$$x_{IRAK4}(t+1) = (\neg x_{MyD88}(t) \wedge x_{TICAM1}(t)) \vee (x_{MyD88}(t))$$

Using maximal reduction, BBE reveals that these two variables are equivalent because also *TICAM1* and *TLR5* are so. The corresponding ODEs after odification are instead given by:

$$x'_{MyD88} = x_{TLR5} - x_{MyD88}$$
$$x'_{IRAK4} = x_{MyD88} + x_{TICAM1} - x_{MyD88} \cdot x_{TICAM1} - x_{IRAK4}$$

where x' denotes the derivative of variable x with respect to time. The ODE variables for *TLR5*, *MyD88*, and *TICAM1* can shown to be still backward equivalent. However, differently from BBE, *IRAK4* is not anymore ODE backward equivalent to the others. Indeed, since ODEs allow a continuous range of values in the interval $[0;1]$, the property that the solution of variables must be equal at all time points must be valid for all possible such values. However, if all variables have value 0.5, then we get derivative with value 0 for *MyD88* and value 0.25 for *IRAK4*, which indeed makes them not ODE backward equivalent.
