# Supplementary material for: Reducing Boolean networks with backward equivalence
Source: BMC Bioinformatics. 2023 May 23;24(Suppl 1):212. doi: 10.1186/s12859-023-05326-9 (PMC10207686; doi:10.1186/s12859-023-05326-9)
Supplement: Supplementary file 2 — Additional file 2: Technical Results. [file 12859_2023_5326_MOESM2_ESM.pdf]

2 Technical Results

2.1 Preliminaries

First, we formalize a BN as follows.

Definition 1 (Boolean Network (BN)) *A BN is a pair (X, F) where $X = \{x_1, ..., x_n\}$ is a set of variables and $F = \{f_{x_1}, ..., f_{x_n}\}$ is a set of update functions, with $f_{x_i} : \mathbb{B}^n \to \mathbb{B}$ being the update function of variable x_i.*

The *state* of a BN is an evaluation of the variables, denoted by a vector of values $\mathbf{s} = (s_{x_1}, \ldots, s_{x_n}) \in \mathbb{B}^n$. Given a *partition of synchronization* $\{K_1, \ldots, K_m\} = \mathcal{K}$ of the variables X, and two states for $\mathbf{s}, \mathbf{t} \in \mathbb{B}^n$, we have a transition $\mathbf{s} \to \mathbf{t}$ if there exists a block K in $\mathcal{K}$ such that

- $t_{x_i} = f_{x_i}(\mathbf{s})$ for all $x_i \in K$
- $t_{x_i} = s_{x_i}$ for all $x_i \notin K$

We next introduce the *state transition graph* of a BN with respect to a given partition of synchronization. This is a graph having all possible states as vertices, and all transition among states as edges.

Definition 2 (State transition graph (STG)) *Let $B = (X, F)$ be a BN and $\mathcal{K}$ a partition of X. The state transition graph of B w.r.t. the synchronization partition $\mathcal{K}$, denoted by $STG_{\mathcal{K}}(B)$, is a pair $(S, T_{\mathcal{K}})$, where $S = \mathbb{B}^n$ is the set vertices, while the set of transitions $T_{\mathcal{K}}$ is defined by*

$$T_{\mathcal{K}} = \{\mathbf{s} \to \mathbf{t} \mid \mathbf{t}_{|K} = F_{|K}(\mathbf{s}) \text{ and } \mathbf{t}_{|X\setminus K} = \mathbf{s}_{|X\setminus K} \text{ for some } \mathbf{s} \in S \text{ and } K \in \mathcal{K}\}.$$

Using common notation, $v_{|I}$ denotes the restriction of a vector v to the set of indices I. When $\mathcal{K}$ is clear from the context or does not have an impact on the statement, we shall drop the subscript $\mathcal{K}$.

We note that $(S, T_{\mathcal{K}})$ corresponds to the STG of a synchronous BN when $\mathcal{K} = \{X\} = \mathcal{K}_{sync}$ and to that of an asynchronous BN when $\mathcal{K} = \{\{x\} \mid x \in X\} = \mathcal{K}_{async}$. The case when $\mathcal{K}$ refines $\mathcal{K}_{sync}$ and is at the same time coarser than $\mathcal{K}_{async}$, instead, describes a middle ground where different sets of variables, the blocks of $\mathcal{K}$, update synchronously within their block, and asynchronously with respect to the other blocks. We call $\mathcal{K}$ *synchronization partition* because the updates of two variables are synchronized if and only if they belong to the same block of $\mathcal{K}$. Notably, this synchronization schema is supported, e.g., by popular BN analysis tools like GINsim [6] under the notion of *priority classes* as described in [7].

We shall use the notation $\mathbf{s} \to^+ \mathbf{t}$ for the transitive closure of the transition relation. With this, we can formally define the notion of attractors.

Definition 3 (Attractor) *Let $B = (X, F)$ be a BN with $STG(B) = (S, T)$. We say that a set of states $A \subseteq S$ is an* attractor *whenever*

1 $\forall \mathbf{s}, \mathbf{s}' \in A$, $\mathbf{s} \to^+ \mathbf{s}'$, and

2 $\forall \mathbf{s} \in A, \forall \mathbf{s}' \in S$, $\mathbf{s} \to^+ \mathbf{s}'$ implies $\mathbf{s}' \in A$.

Attractors are hence absorbing strongly connected components in the STG. An attractor A such that $|A| = 1$ is called a *steady state* (also named *point attractor*). We also denote with $|A|$ the *length* of attractor A.

2.2 Boolean Backward Equivalence

Let X be a set, and $\mathcal{H}$ a partition over it. Any partition obtained by breaking down the blocks of $\mathcal{H}$ into sub-blocks is said to be a refinement of $\mathcal{H}$. The notion of BBE, the algorithm for its computation, and the notion of BN reduced up to a BBE do not depend on the used synchronization partition $\mathcal{K}$. However, as we shall see, a BBE $\mathcal{H}$ guarantees the preservation of dynamics of a BN only if $\mathcal{H}$ refines $\mathcal{K}$. This can be guaranteed by using as initial partition $\mathcal{G}$ either $\mathcal{K}$, or any refinement of it.

We first introduce the notion of *constant* state on a partition $\mathcal{H}$.

Definition 4 (Constant State) *Let X be a set of variables, and $\mathcal{H}$ a partition of X. A state $\mathbf{s} \in \mathbb{B}^n$ is* constant *on $\mathcal{H}$ if and only if for all $H \in \mathcal{H}$ and $x_i, x_j \in H$ it holds that $s_{x_i} = s_{x_j}$.*

We now define the notion of BN reduced up to a BBE $\mathcal{H}$. Each variable in the reduced BN represents one block of $\mathcal{H}$. Informally, we pick one variable per block, select the update function of any variable in such block and replace all variables in it with the representative of the block the variable belongs to. Formally, we denote by $f[a/b]$ the term arising by replacing each occurrence of a by b in the function f.

Definition 5 (BN reduction) *The reduction of B up to $\mathcal{H}$, denoted by $B_{\mathcal{H}}$, is the BN $(X_{\mathcal{H}}, F_{\mathcal{H}})$ where $F_{\mathcal{H}} = \{f_{x_H} \mid H \in X_{\mathcal{H}}\}$ and, for any $H \in \mathcal{H}$ and some $x_k \in H$, one sets*
$f_{x_H} = f_{x_k}\left[x_i/x_{H'} \mid \forall H' \in X_{\mathcal{H}}, \forall x_i \in H'\right]$.

Algorithm S1: Compute maximal BBE of (X, F) refining an initial partition $\mathcal{G}$

Result: maximal BBE $\mathcal{H}$ that refines an arbitrary partition $\mathcal{G}$
$\mathcal{H} \leftarrow \mathcal{G}$;
while *true* **do**
  if $\Phi^{\mathcal{H}}$ *is valid* **then**
    return $\mathcal{H}$;
  else
    s $\leftarrow$ get a state that satisfies $\neg\Phi^{\mathcal{H}}$;
    $\mathcal{H}' \leftarrow \emptyset$;
    for $H \in \mathcal{H}$ **do**
      $H_0 = \{x_i \in H : f_{x_i}(\mathbf{s}) = 0\}$;
      $H_1 = \{x_i \in H : f_{x_i}(\mathbf{s}) = 1\}$;
      $\mathcal{H}' = \mathcal{H}' \cup \{H_1\} \cup \{H_0\}$;
    end
    $\mathcal{H} \leftarrow \mathcal{H}' \setminus \{\emptyset\}$;
  end
end

The partition refinement algorithm is shown in Algorithm S1. Its inputs are a BN and $\mathcal{G}$, an initial partition of its variables X. The output of the algorithm is the coarsest partition that is a BBE and that refines $\mathcal{G}$.
The number of iterations needed to reach a BBE depends on the state assignments that the SAT solver provides but is at most $|X| = n$ because a partition over X can be refined at most $|X|$ times. Each iteration requires to solve a SAT problem which is known to be NP-complete [48]. However, as discussed in the main text, our implementation can scale to the largest models present in popular BN repositories.
We first show that given an initial partition there exists a unique coarsest BBE.

Theorem 1 *Fix a BN (X, F) and a partition $\mathcal{G}$. There exists a unique maximal BBE $\mathcal{H}$ that refines $\mathcal{G}$.*

Proof of Theorem 1 Let $\mathcal{H}_1$, $\mathcal{H}_2$ be two BBE partitions that refine some other partition $\mathcal{G}$ that is not necessarily a BBE. Let R_1, R_2, R_3 be equivalence relations over X inducing $\mathcal{H}_1, \mathcal{H}_2$ and $\mathcal{G}$, respectively. We start by noting that $R = (R_1 \cup R_2)^* \subseteq R_3$, where the asterisk denotes the transitive closure. Hence, $\mathcal{X}_R$ is a refinement of $\mathcal{G}$, where $\mathcal{X}_R = X/R$. We next show that $\mathcal{X}_R$ is a BBE partition. To this end, fix some $\mathbf{s} \in \mathbb{B}^n$ that is constant on $\mathcal{X}_R$. Since $R_i \subseteq R$, this implies that $\mathbf{s}$ is constant on $\mathcal{X}_i$ which, in virtue of $\mathcal{H}_i$ being a BBE, implies that $F(\mathbf{s}) \in \mathbb{B}^n$ is constant on $\mathcal{H}_i$. This implies that $F(\mathbf{s}) \in \mathbb{B}^n$ is constant on $\mathcal{X}_R$, i.e., that $\mathcal{X}_R$ is indeed a BBE partition. The overall claim follows by noting that the finiteness of X implies that there are finitely many BBE partitions $\mathcal{H}_i$ that refine any given partition $\mathcal{G}$ of X. □

We now prove that Algorithm S1 provides indeed the maximal BBE that refines the initial one.

Theorem 2 *Algorithm S1 computes the maximal BBE partition refining $\mathcal{G}$.*

Proof of Theorem 2 Assume that $\mathcal{G}'$ denotes the coarsest BBE partition that refines some given partition $\mathcal{G}$. Set $\mathcal{H}_0 := \mathcal{G}$ and define for all $k \geq 0$

$$\mathcal{H}_{k+1} := \big(\{H_0 \mid H \in \mathcal{H}_k\} \cup \{H_1 \mid H \in \mathcal{H}_k\}\big) \setminus \{\emptyset\},$$

where H_0 and H_1 are as in Algorithm S1. Then, a proof by induction over $k \geq 1$ shows that (a) $\mathcal{G}'$ is a refinement of $\mathcal{H}_k$ and (b) $\mathcal{H}_k$ is a refinement of $\mathcal{H}_{k-1}$, for all $k \geq 1$. Since $\mathcal{G}'$ is a refinement of any $\mathcal{H}_k$, it holds that $\mathcal{G}' = \mathcal{H}_k$ if $\mathcal{H}_k$ is a BBE partition. Since X is finite, b) allows us to fix the smallest $k \geq 1$ such that $\mathcal{H}_k = \mathcal{H}_{k-1}$. This, in turn, implies that $\mathcal{H}_{k-1}$ is a BBE. □

2.3 Relating Dynamics of Original and Reduced BNs

We next relate the STGs of the original and the reduced BN.

Definition 6 *Fix a BN $B = (X, F)$, a BBE $\mathcal{H}$ of B, a synchronization partition $\mathcal{K}$, and $STG_{\mathcal{K}}(B) = (S, T_{\mathcal{K}})$ such that $\mathcal{K}$ is coarser than $\mathcal{H}$. With this, the STG of $B/\mathcal{H} = (X_{\mathcal{H}}, F_{\mathcal{H}})$ has synchronization partition $\mathcal{K}_{\mathcal{H}} = \big\{\{H_j \mid x_i \in K \text{ and } x_i \in H_j\} \mid K \in \mathcal{K}\big\}$ and states $m_{\mathcal{H}}(S_{|\mathcal{H}})$, where*

- *$S_{|\mathcal{H}}$ denotes all states of S constant on $\mathcal{H}$ and;*

- $m_{\mathcal{H}} : S_{|\mathcal{H}} \to S_{\mathcal{H}}$ *is given by* $m_{\mathcal{H}}(\boldsymbol{s}) = (v_{H_1}, \ldots, v_{H_{|\mathcal{H}|}})$ *and extends to sets via elementwise application, while* $v_{H_j} := s_{x_i}$ *for previously chosen representative* $x_i \in H_j$.

The following lemma ensures that all attractors of $STG_{\mathcal{K}}(B)$ containing states constant on $\mathcal{H}$ are preserved by $STG_{\mathcal{K}_{\mathcal{H}}}(B/\mathcal{H})$.

Lemma 3 (Constant attractors) *Fix a BN* $B = (X, F)$, *a BBE* $\mathcal{H}$ *of* B *and* $STG_{\mathcal{K}}(B) = (S, T_{\mathcal{K}})$ *such that* $\mathcal{K}$ *is coarser than* $\mathcal{H}$. *Let us further assume that* A *is an attractor of* $STG_{\mathcal{K}}(B)$. *With this, if* $A \cap S_{|\mathcal{H}} \neq \emptyset$, *then* $A \subseteq S_{|\mathcal{H}}$.

Proof of Lemma 3 By assumption, we can pick a state $\mathbf{s} \in A$ that is constant on $\mathcal{H}$. The fact that $\mathcal{H}$ is a BBE refining $\mathcal{K}$ ensures that any state $\mathbf{t}$ with $\mathbf{s} \to^{+} \mathbf{t}$ is also constant on $\mathcal{H}$. Actually, it is trivial to show that $A = \{\mathbf{t} \mid \mathbf{s} \to^{+} \mathbf{t}\}$, thus implying that $A \subseteq S_{|\mathcal{H}}$. □

The next proposition ensures that BBE does not generate spurious trajectories or attractors in the reduced system. In particular we show that the STG of the reduced BN is a subgraph (modulo state renaming) of the STG of the original BN.

Proposition 4 (Reduction isomorphism) *Fix a BN* $B = (X, F)$, *a BBE* $\mathcal{H}$ *of* B *and* $STG_{\mathcal{K}}(B) = (S, T_{\mathcal{K}})$ *such that* $\mathcal{K}$ *is coarser than* $\mathcal{H}$. *It can be shown that* $STG_{\mathcal{K}_{\mathcal{H}}}(B/\mathcal{H})$ *is described by* $\big(m_{\mathcal{H}}(S_{|\mathcal{H}}), m_{\mathcal{H}}(T_{\mathcal{K}} \cap (S_{|\mathcal{H}} \times S_{|\mathcal{H}})\big)$. *Furthermore*

1. *For all states* $\boldsymbol{s} \in S_{|\mathcal{H}}$ *it holds* $F_{\mathcal{H}}(m_{\mathcal{H}}(\boldsymbol{s})) = m_{\mathcal{H}}(F(\boldsymbol{s}))$.
2. *For all states* $\boldsymbol{s} \in S_{\mathcal{H}}$ *it holds* $F(m_{\mathcal{H}}^{-1}(\boldsymbol{s})) = m_{\mathcal{H}}^{-1}(F_{\mathcal{H}}(\boldsymbol{s}))$.

Proof of Proposition 4 Follows readily from the definition of a BBE, $STG_{\mathcal{K}_{\mathcal{H}}}(B/\mathcal{H})$, and $m_{\mathcal{H}}$. □

Instead, the following example shows that it is necessary for the initial partition to be a refinement of the synchronization partition of the model.

Example 1 *Let us consider the 3-variables example from Fig. 1. Let us assume that the model is equipped with the synchronization partition* $\mathcal{K} = \{\{x_1\}, \{x_2, x_3\}\}$. *This means, e.g., that from state* 000 *we can go either in state* 100 *by updating* x_1, *or in state* 010. *From both states, we can go to state* 110. *If we apply BBE using the initial partition* $\mathcal{H} = \{\{x_1, x_2, x_3\}\}$ *that does not refine* $\mathcal{K}$, *we get the same reduced model as in Fig. 1. In such reduced model, we find the reduced variable* $x_{1,2}$ *representing variables* x_1 *and* x_2 *which, however, shall not be updated synchronously according to* $\mathcal{K}$. *Therefore, it is not possible to define the synchronization partition* $\mathcal{K}_{\mathcal{H}}$ *as given in Definition 6. Note furthermore that if we opt for a synchronization partition enabling the synchronous update of* $x_{1,2}$ *and* x_3, *we get the STG from the top-right of Fig. 1. Here, our reduction isomorphism result does not hold, because the reduced STG cannot express the above-discussed 2-steps path from 000 to 110. In fact, the corresponding path from 00 to 10 is done in only 1 transition.*

We can now state the main result of our approach, namely that the BBE reduction of a BN for a BBE $\mathcal{H}$ exactly preserves all attractors that are constant on $\mathcal{H}$ up to renaming with $m_{\mathcal{H}}$.

Theorem 5 (Constant attractor preservation) *Fix a BN* $B = (X, F)$, *a BBE* $\mathcal{H}$ *of* B *and* $STG_{\mathcal{K}}(B) = (S, T_{\mathcal{K}})$ *such that* $\mathcal{K}$ *is coarser than* $\mathcal{H}$. *Let us further assume that* A *is an attractor of* $STG_{\mathcal{K}}(B)$. *With this, if* $A \cap S_{|\mathcal{H}} \neq \emptyset$, *then* $m_{\mathcal{H}}(A)$ *is an attractor of* $STG_{\mathcal{K}}(B)$. *Furthermore, given a state* $\boldsymbol{s} \in S_{|\mathcal{H}}$ *and an attractor* A *such that* $A \cap S_{|\mathcal{H}} \neq \emptyset$, *we have that* A *is reachable from* $\boldsymbol{s}$ *if and only if* $m_{\mathcal{H}}(A)$ *is reachable from* $m_{\mathcal{H}}(\boldsymbol{s})$.

Proof of Theorem 5 The theorem readily follows from Lemma 3 and Proposition 4. □
