# Supplementary material for: Reducing Boolean networks with backward equivalence
Source: BMC Bioinformatics. 2023 May 23;24(Suppl 1):212. doi: 10.1186/s12859-023-05326-9 (PMC10207686; doi:10.1186/s12859-023-05326-9)
Supplement: Supplementary file 3 — Additional file 3: Application of BBE to a BN with partially asynchronous schema. [file 12859_2023_5326_MOESM3_ESM.pdf]

3 Application of BBE to a BN with partially asynchronous schema

We present an application of BBE to a BN with partially asynchronous schema. According to Additional file 2, here we use a partition of synchronisation $\mathcal{K}$ separating variables in blocks. At each time point, one block $K \in \mathcal{K}$ is non-deterministically selected, and all and only the variables in K are updated syncrhonously. As mentioned in previous sections, this type of synchronization schema is supported, e.g., by popular BN analysis tools like GINsim [6] under the notion of *"priority classes"* [7]. We focus on the BN of [46] which is displayed in the left part of Fig. S1. The BN models neurogenesis: the process by which nervous system cells, the neurons, are produced by neural stem cells.

$$
\begin{aligned}
x_{Her6}(t+1) &= \neg x_{miR9}(t) \wedge \neg x_N(t) \\
x_{HuC}(t+1) &= \neg x_{miR9}(t) \wedge \neg x_P(t) \\
x_N(t+1) &= x_{HuC}(t) \\
x_P(t+1) &= x_{Her6}(t) \vee x_{Zic5}(t) \\
x_{Zic5}(t+1) &= \neg x_{miR9}(t) \wedge \neg x_N(t) \\
x_{miR9}(t+1) &= \neg x_{Her6}(t) \wedge \neg x_N(t)
\end{aligned}
$$

$$
\begin{aligned}
x_{\{Her6,Zic5\}}(t+1) &= \neg x_{\{miR9\}}(t) \wedge \neg x_{\{N\}}(t) \\
x_{\{HuC\}}(t+1) &= \neg x_{\{miR9\}}(t) \wedge \neg x_{\{P\}}(t) \\
x_{\{N\}}(t+1) &= x_{\{HuC\}}(t) \\
x_{\{P\}}(t+1) &= x_{\{Her6,Zic5\}}(t) \\
x_{\{miR9\}}(t+1) &= \neg x_{\{Her6,Zic5\}}(t) \wedge \neg x_{\{N\}}(t)
\end{aligned}
$$

Figure S1: (Left) The variables and update functions. (Right) The reduced BN obtained after collapsing the variables x_{Her6}, x_{Zic5} into one single variable component $x_{\{Her6,Zic5\}}$.

Hypothesis. The authors consider a fully synchronous schema wherein all variables are updated at the same time. However, the set $\{x_{Her6}, x_{Zic5}, x_P\}$ seems to update synchronously in vivo, while x_{miR9}, x_{HuC} and x_N update asynchronously both with the set $\{x_{Her6}, x_{Zic5}, x_P\}$, and with each other [46],

Configuration. To this end, we create a corresponding partition of synchronization as follows:

$$\mathcal{K} = \{\{x_{Her6}, x_{Zic5}, x_P\}, \{x_{miR9}\}, \{x_{HuC}\}, \{x_N\}\},$$

The STG according to this partition of synchronization is given in Fig. S2. This STG has been obtained using the GINsim tool.
We reduce this model using BBE. In order to be coherent with our theory, we set $\mathcal{K}$ as the initial partition for our reduction algorithm Algorithm S1. This enables us to use the results on preservation of dynamics from Additional file 2.

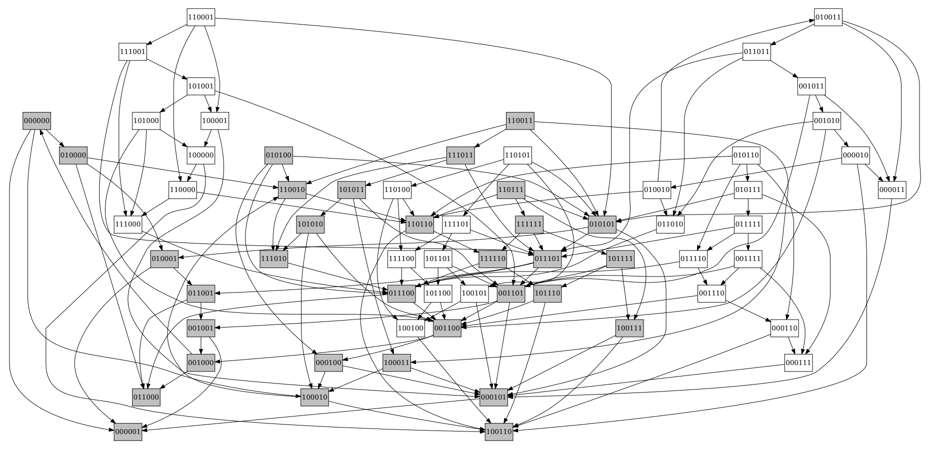

Figure S2: The STG of the BN of Fig. S1 (left) according to the partition of synchronization $\mathcal{K} = \{\{x_{Her6}, x_{Zic5}, x_P\}, \{x_{miR9}\}, \{x_{HuC}\}, \{x_N\}\}$.

Results. The resulting BBE is:

$$\{\{x_{Her6}, x_{Zic5}\}, \{x_P\}, \{x_{miR9}\}, \{x_{HuC}\}, \{x_N\}\}$$

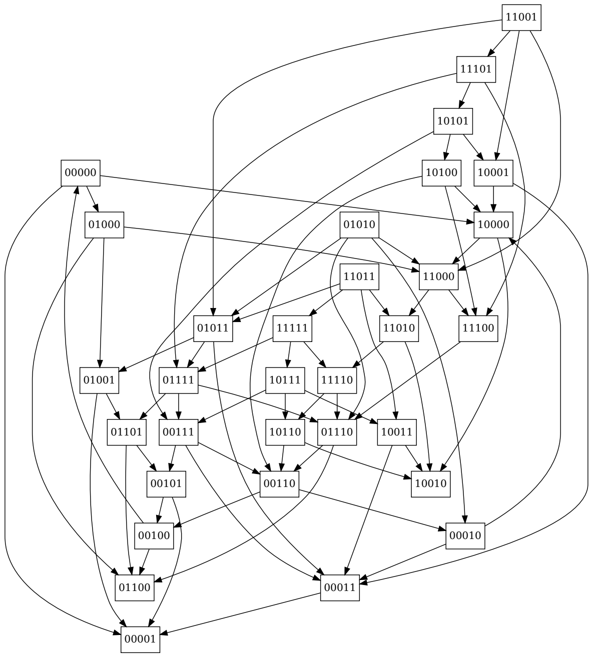

Figure S3: The STG of the BBE-reduced BN. This STG corresponds to the original one (Fig. S2) after dropping all states non constant on the used BBE. Note that the first and the fifth digit of each preserved state are collapsed into one single digit (these two digits are always equal in the preserved states).

Please note that, in this specific example, the update functions of the two related variables (x_{Her6}, and x_{Zic5}) have the same update function. However, we have seen in other examples here that BBE might relate also variables with apparently unrelated update functions (e.g., the case of x_{MyD88} and x_{IRAK4} in Additional file 1).
The grey states of Fig. S2 correspond to the constant states of this BBE partition and, consequently, these are the preserved states after reduction. We obtain the reduced BN by collapsing $\{x_{Her6}, x_{Zic5}\}$ into a single variable component represented by the variable $x_{\{Her6,Zic5\}}$. The reduced BN is displayed in the right part of Fig. S1.

Interpretation and Discussion. The STG of the reduced BN is displayed in Fig. S3. The reduction isomorphism (Proposition 4) guarantees that the constant states are preserved with all the transitions between them, and that there are no transitions from the constant states to the non-constant states. According to Theorem 5, constant attractors are preserved (i.e., attractors containing at least one state constant on the BBE). In this case, the original BN has 3 steady states (the states 011000, 000001 and 100110) and all of them are preserved in the reduced BN.
