# Supplementary material for: Reducing Boolean networks with backward equivalence
Source: BMC Bioinformatics. 2023 May 23;24(Suppl 1):212. doi: 10.1186/s12859-023-05326-9 (PMC10207686; doi:10.1186/s12859-023-05326-9)
Supplement: Supplementary file 4 — Additional file 4: An application of BBE to randomly generated Boolean Networks. [file 12859_2023_5326_MOESM4_ESM.pdf]

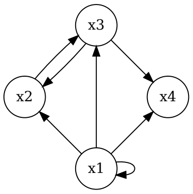

$$\begin{aligned} x_1(t+1) &= x_1(t) \\ x_2(t+1) &= x_1(t) \vee x_3(t) \\ x_3(t+1) &= x_1(t) \wedge x_2(t) \\ x_4(t+1) &= \neg x_1(t) \wedge x_3(t) \end{aligned}$$

Figure S4: (Left) A randomly generated graph of influences for $n = 4, k = 2$. (Right) A BN with randomly generated update functions coherent with (Left).

4 Application of BBE to randomly generated Boolean Networks

In this section, we apply BBE to randomly generated BNs. These have been constructed by using an n-k model [49] as described in Kauffman's seminal work on BNs [1]. In partiuclar, n refers to the number of variables in the generated BNs, while k to the number of incoming influences of each variable. The process is described in Fig. S4: we first obtain a directed graph on n nodes. For each node, the number of incoming edges is drawn randomly from a Poisson distribution with mean k, choosing the source nodes randomly (see left part of Fig. S4). On average, the nodes of such randomly generated BNs will have k incoming edges. The nodes are then transformed in BN variables by using a procedure specified in [49] to randomly generate update functions coherent with the previously generated graph of influences (right part of Fig. S4). The procedure is implemented in the R package BoolNet [49]. In what comes later we will study BNs generated by varying both n (size of the BN) and k (*density* of the BN). For the additional parameters of the package not mentioned here, we use default values from [49].

Purpose. Our purpose is to investigate the scalability of BBE to randomly generated BNs as the number of variables increases, and estimate the expected loss of attractors. We consider two different values for k: 2, and 1, studying BBE at the varying of the density of influences in the BNs. [4]

Configuration. For $k = 2$, we generate *100* BNs for $n = 50$, $n = 100$, and $n = 200$ variables, resulting in *300* BNs overall. As done in the main text, we reduce these BNs using maximal and IS initial partitions, and compute the reduction ratios (paragraph *"Results on Reduction Magnitude"*). We also compute the number of attractors in the original and reduced BNs (paragraph *"Results related to attractor preservation."*). We then repeat the same analysis for $k = 1$, considering *300* more BNs. Overall, we consider 600 randomly generated BNs.

Results on Reduction Magnitude. As a reminder, the reduction ratio is defined as the fraction of the number of variables in the reduced BN, over the number of variables in the original BN. We display the reduction ratio for these *300* BNs for varying size and $k = 2$ in Fig S5. Both scenarios (IS and maximal) lead to the reduction of 299 out of the 300 BNs considered. Only one BN with $n = 50$ was not reduced (for any of the two initial partitions). When the red dot and the blue cross coincide, the IS and the maximal reduction have the same reduction ratio. Fig. S6 displays the same analysis for 300 networks of $k = 1$. In Table S1, we present the average reduction ratios of the BNs obtained for the different values of n and k. We can see that models generated for $k = 1$ allow for stronger reductions. We interpret this as follows: the more *sparse* is a BN (i.e., the less influences there are among the variables), the more effective becomes BBE.

	IS	*Maximal*	*IS*	*Maximal*
	k=2		k=1	
n=50	*0.878*	*0.875*	*0.556*	*0.542*
n=100	*0.856*	*0.852*	*0.517*	*0.502*
n=200	*0.837*	*0.833*	*0.464*	*0.450*

Table S1: Mean IS and maximal reduction ratio of the 600 randomly generated BNs.

In Table S2 (left) we provide the average reduction time, for IS and maximal initial partitions, of the 600 randomly generated BNs. We observe that the average reduction time seems to increase linearly with the size of the considered BN. In Table S2 (right) we display the maximum reduction time; in the worst case scenario the BBE-reduction was performed in about 3 seconds. The IS and maximal reductions seem to take about the same time.

Results related to attractor preservation. For $k = 2$, and $n = 100$ and 200, the tool BNS failed several times due to time-out (we imposed an arbitrary time-out of 30 minutes). Therefore, we focus only on the BNs with

[4] We chose $k = 2$ as maximum value because it was the largest value used in a similar study for a different analysis technique in [12] (section 6.2). In [12], further used values for k were 1.9, 1.889, and 1.875. Here we preferred to use $k = 1$ because we are interested in studying the effect on BBE of higher changes in the density of the interaction graph.

Average	*IS*	*Maximal*	*IS*	*Maximal*
	k=2		k=1	
n=50	*0.591*	*0.614*	*0.442*	*0.477*
n=100	*1.402*	*1.428*	*0.989*	*1.034*
n=200	*2.433*	*2.489*	*1.869*	*1.894*

Maximum	*IS*	*Maximal*	*IS*	*Maximal*
	k=2		k=1	
n=50	*0.870*	*0.943*	*0.923*	*0.901*
n=100	*1.888*	*1.907*	*1.437*	*1.478*
n=200	*3.285*	*3, 363*	*3.224*	*2.571*

Table S2: Mean (Left) and maximum (Right) reduction time of the 600 randomly generated BNs for IS and maximal initial partitions.

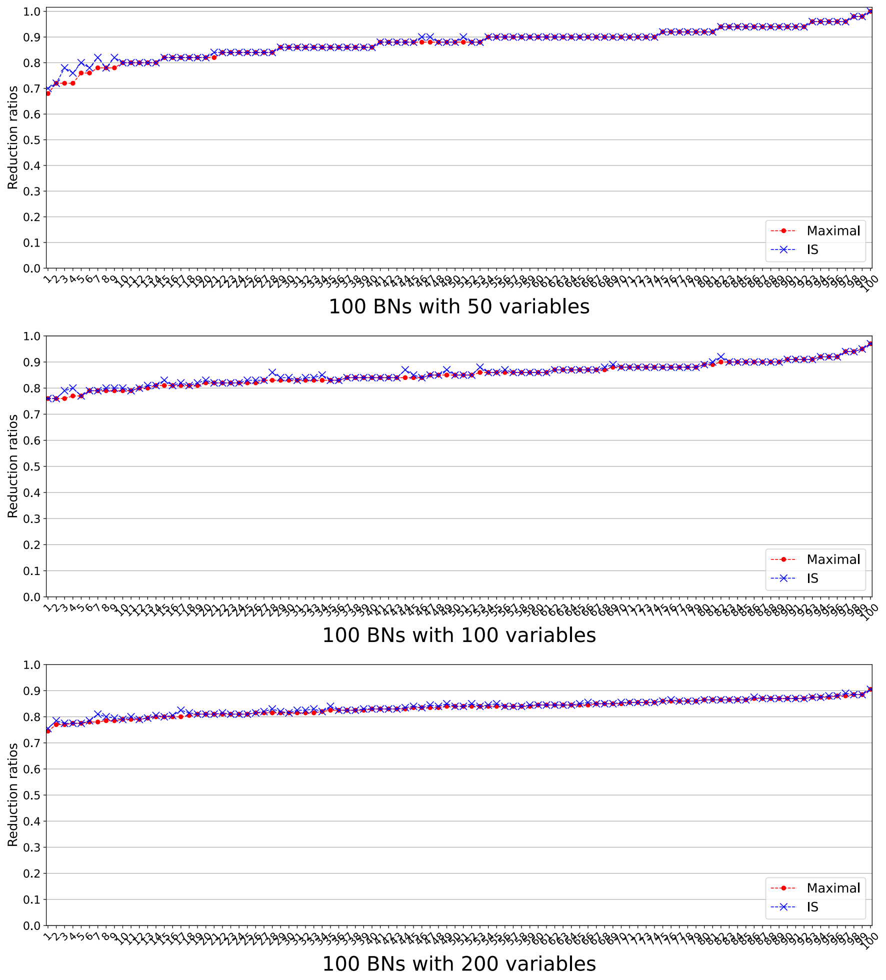

Figure S5: Reduction ratios for *300* randomly generated BNs using $k = 2$.

$n = 50$ for which we experienced only two time-outs (models 44 and 46 for which we report 10^{-1} as number of obtained attractors to stress that attractor generation failed). We consider only the *99* BNs that admitted BBE reduction. We display in Fig. S7 (top) the number of attractors in the original, the IS, and the maximal reduced BNs. In most cases, BBE preserves all attractors; the cases wherein attractors are lost are these where the orange line is above the other two lines (see, e.g., the BNs 7 and 55). The IS and maximal reduction scenario seemed to preserve the same number of attractors; these are cases wherein the red dot and the blue cross coincide. However, this case is not general as, for instance, BN 15 wherein the IS reduction preserves more attractors than the maximal one. The bottom part of Fig. S7 displays the corresponding information in the case of real BNs. Given the better reduction ratio obtained for the real models, here we tend to preserve a lower percentage of attractors. In $k = 1$, attractors generation succeeded always within the specified time limit of 30 minutes. The results are displayed in Fig. S8. Surprisingly, the much better reduction ratio than case $k = 2$ does not lead to lower preservation of attractors. Attractors are often fully preserved.

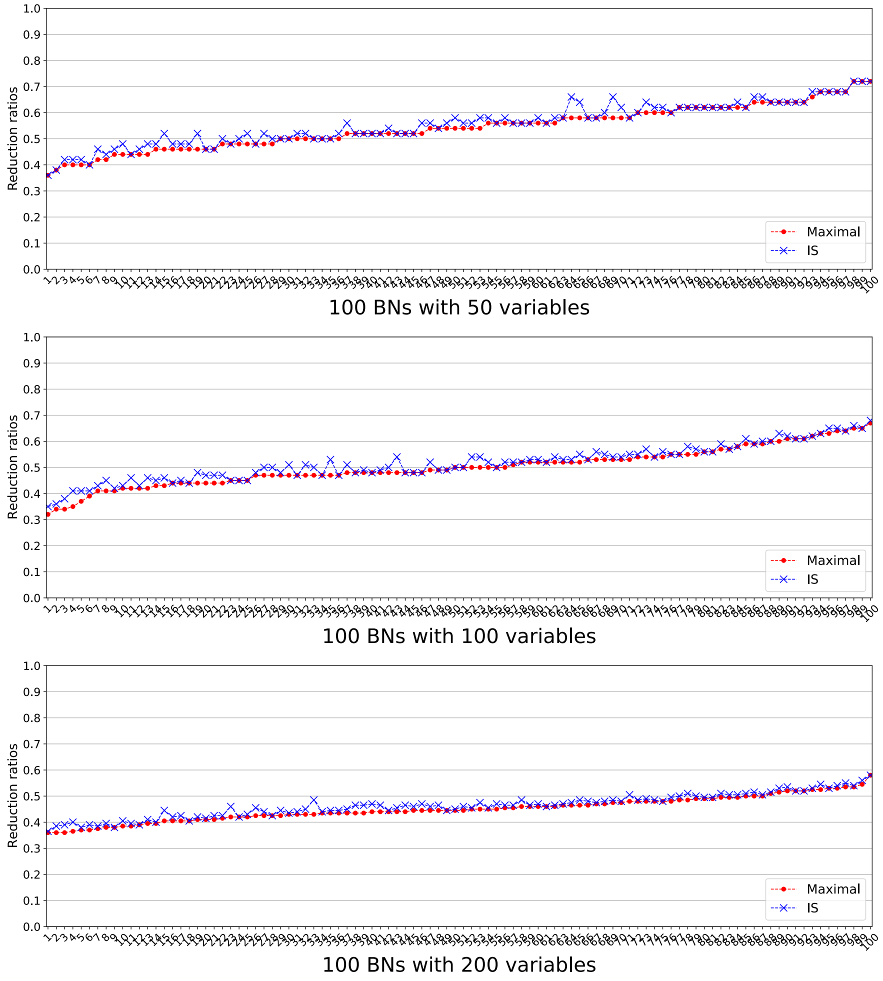

Figure S6: Reduction ratios for *300* randomly generated BNs using $k = 1$.

Interpretation. From Table S1, we see that BBE scales well with the size of BNs: while the number of variables increases, the reduction ratio decreases. Indeed, for $k = 2$ the average IS reduction ratio goes from 0.88 for $n = 50$, to 0.83 for $n = 200$. The same behavior is observed in the case of maximal reduction, and also in both cases for $k = 1$. The same table shows that the average reduction ratio is better when $k = 1$, meaning that BBE performs better when the density of the interaction graph of a BN (see Fig. S4, left) is low.

In the case of randomly generated BNs, attractors are often fully preserved. This is in contrast with the realistic BNs from the repositories. For $k = 2$ and $n = \mathit{100}$ and $n = \mathit{200}$, the BNS tool fails to compute the attractors within the 30-minutes time-out arbitrarily chosen by us. Here, several variables might have a high number of incoming influences. This leads to complex update Boolean functions that the BNS tool fails to manage. Instead, BBE terminated correctly on all randomly generated BNs in less than 3.5 seconds in all cases.

In Figs. S7, and S8 we have seen that for randomly generated BNs, BBE tends to preserve more attractors than for realistic BNs from the repositories. This might be reasonable for the cases $k = 2$, for which we have higher reduction ratios (we reduce less) than for the realistic BNs. Instead, this is somehow surprising for $k = 1$ where we reduce more. Given the persistent result obtained for $k = 2$ and $k = 1$ (and given that, as discussed, we considered a larger interval for k than in [12]), we suspect that this depends on oher parameters of the generation process not considered in our study. For example, a possible interpretation is that by looking at Figs. S7 and S8 we can see that the BNs from the repositories can generate more attractors (up to 10^5) than the randomly generated BNs (slightly above 10^2). However, we believe that a deeper study on the used generation process from the R package BoolNet is out of the scope of this paper.

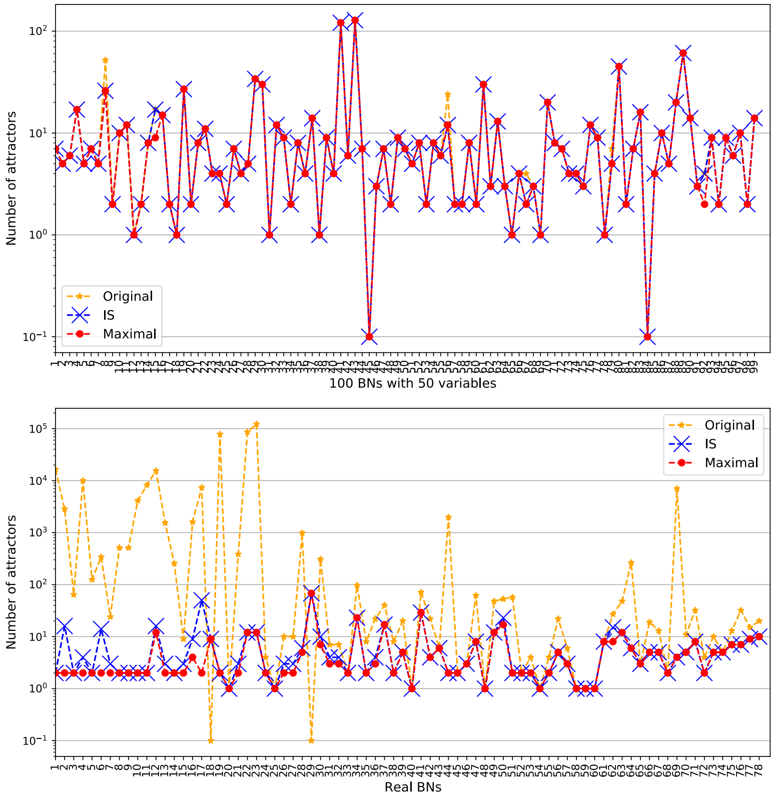

Figure S7: (Top) number of preserved attractors for 100 networks with 50 variables and $k = 2$. (Bottom) number of preserved attractors for the realistic BNs of Table S5 from the two online repositories. In bottom, for models 17 and 28 we provide "0 attractors" in the *Original* case to stress that it was not possible to compute the attractors for the original models 17 and 28 (while it was possible for their reductions). The number of attractors are given in log scale.

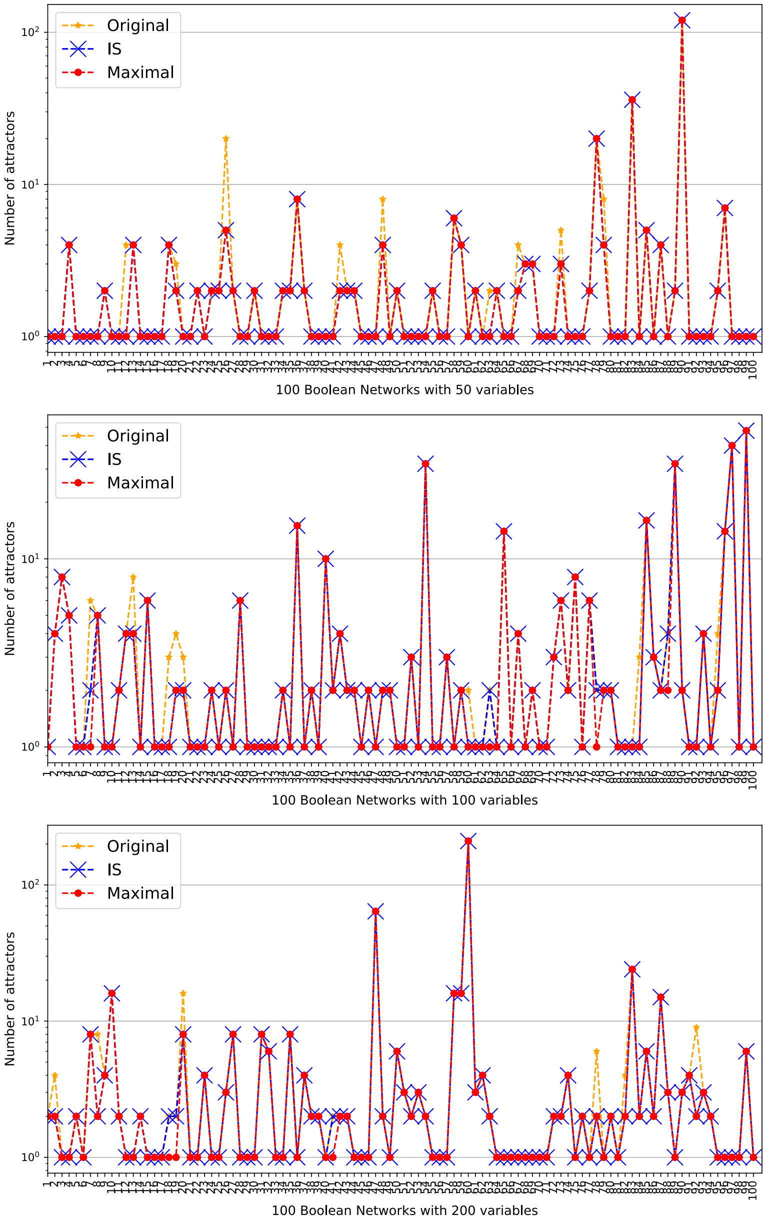

Figure S8: Number of preserved attractors for 300 randomly generated BNs of different sizes. In the generation process we set $k = 1$.
