# Supplementary material for: Reducing Boolean networks with backward equivalence
Source: BMC Bioinformatics. 2023 May 23;24(Suppl 1):212. doi: 10.1186/s12859-023-05326-9 (PMC10207686; doi:10.1186/s12859-023-05326-9)
Supplement: Supplementary file 5 — Additional file 5: Tables with detailed results from large-scale experiments. [file 12859_2023_5326_MOESM5_ESM.pdf]

5 Tables

Model ID	URL	Size			Reduction Ratio	
		N	N_i	N_m	R_i	R_m
1 [50]	ginsim.org/node/97	26	7	4	26.92	15.38
2 [51]	www.ebi.ac.uk/biomodels/MODEL1606020000	19	11	3	57.89	15.79
3 [50]	ginsim.org/node/126	24	11	4	45.83	16.67
4 [52]	ginsim.org/model/drosophila_mesoderm	57	34	11	59.65	19.3
5 [53]	www.ebi.ac.uk/biomodels/BIOMD0000000592	10	2	2	20.0	20.0
6 [53]	www.ebi.ac.uk/biomodels/BIOMD0000000593	18	13	4	72.22	22.22
7 [54]	ginsim.org/model/eggshell_patterning	8	4	2	50.0	25.0
8 [50]	ginsim.org/node/115	16	8	5	50.0	31.25
9 [50]	ginsim.org/node/102	23	13	8	56.52	34.78
10 [50]	ginsim.org/node/109	19	8	7	42.11	36.84
11 [50]	ginsim.org/node/144	24	11	9	45.83	37.5
12 [55]	ginsim.org/model/sex_determination_mammals	37	16	14	43.24	37.84
13 [50]	ginsim.org/node/96	34	23	15	67.65	44.12
14 [50]	ginsim.org/node/160	18	11	8	61.11	44.44
15 [56]	ginsim.org/node/183	30	25	14	83.33	46.67
16 [54]	ginsim.org/model/eggshell_patterning	24	15	12	62.5	50.0
17 [57]	www.ebi.ac.uk/biomodels/MODEL2101150001	92	72	46	78.26	50.0
18 [58]	ginsim.org/node/185	103	54	52	52.43	50.49
19 [59]	ginsim.org/model/immune-checkpoints	66	43	35	65.15	53.03
20 [60]	www.ebi.ac.uk/biomodels/MODEL1506260002	45	24	24	53.33	53.33
21 [50]	ginsim.org/node/89	18	11	10	61.11	55.56
22 [61]	ginsim.org/node/79	36	23	21	63.89	58.33
23 [61]	ginsim.org/node/79	71	44	42	61.97	59.15
24 [62]	ginsim.org/model/monocytes-to-dc	96	81	57	84.38	59.38
25 [63]	www.ebi.ac.uk/biomodels/MODEL1506260000	82	49	49	59.76	59.76
26 [3]	www.ebi.ac.uk/biomodels/MODEL1504170000	10	7	6	70.0	60.0
27 [3]	www.ebi.ac.uk/biomodels/MODEL1504170003	10	7	6	70.0	60.0
28 [64]	ginsim.org/node/236	41	27	25	65.85	60.98
29 [65]	ginsim.org/model/tcell-checkpoint-inhibitors-tcla4-pd1	218	140	136	64.22	62.39
30 [55]	ginsim.org/model/sex_determination_mammals	19	14	12	73.68	63.16
31 [3]	www.ebi.ac.uk/biomodels/MODEL1504170002	9	7	6	77.78	66.67
32 [3]	www.ebi.ac.uk/biomodels/MODEL1504170001	9	7	6	77.78	66.67
33 [66]	ginsim.org/node/26	12	12	8	100.0	66.67
34 [30]	ginsim.org/node/225	42	37	29	88.1	69.05
35 [67]	ginsim.org/node/227	28	22	20	78.57	71.43
36 [31]	ginsim.org/node/78	40	29	29	72.5	72.5
37 [68]	ginsim.org/node/180	48	38	35	79.17	72.92
38 [45]	ginsim.org/node/173	53	41	39	77.36	73.58
39 [30]	ginsim.org/node/225	128	103	95	80.47	74.22
40 [45]	ginsim.org/node/173	16	12	12	75.0	75.0
41 [69]	ginsim.org/node/39	20	15	15	75.0	75.0
42 [67]	ginsim.org/node/227	33	26	25	78.79	75.76
43 [45]	ginsim.org/node/173	17	13	13	76.47	76.47
44 [70]	www.ebi.ac.uk/biomodels/MODEL1305240000	30	23	23	76.67	76.67
45 [71]	www.ebi.ac.uk/biomodels/MODEL2004040001	56	44	43	78.57	76.79
46 [72]	www.ebi.ac.uk/biomodels/BIOMD0000000562	28	22	22	78.57	78.57
47 [66]	ginsim.org/node/25	39	31	31	79.49	79.49
48 [73]	ginsim.org/node/188	35	31	28	88.57	80.0
49 [74]	ginsim.org/node/69	10	8	8	80.0	80.0
50 [75]	ginsim.org/model/sex_determination_chicken	10	8	8	80.0	80.0
51 [76]	www.ebi.ac.uk/biomodels/MODEL1610060000	31	26	25	83.87	80.65
52 [77]	ginsim.org/node/248	37	32	30	86.49	81.08
53 [66]	ginsim.org/node/29	16	13	13	81.25	81.25
54 [50]	ginsim.org/node/152	11	9	9	81.82	81.82
55 [66]	ginsim.org/node/21	50	41	41	82.0	82.0
56 [30]	ginsim.org/node/225	110	91	91	82.73	82.73
57 [45]	ginsim.org/node/173	18	15	15	83.33	83.33
58 [78]	ginsim.org/node/50	6	6	5	100.0	83.33
59 [79]	ginsim.org/model/HSPC_MSC	26	23	22	88.46	84.62
60 [80]	ginsim.org/node/82	7	6	6	85.71	85.71
61 [80]	ginsim.org/node/82	7	6	6	85.71	85.71
62 [81]	ginsim.org/node/31	14	12	12	85.71	85.71
63 [82]	ginsim.org/node/41	21	18	18	85.71	85.71
64 [76]	www.ebi.ac.uk/biomodels/MODEL1610060001	29	27	25	93.1	86.21
65 [75]	ginsim.org/model/sex_determination_chicken	15	13	13	86.67	86.67
66 [20]	ginsim.org/node/87	60	52	52	86.67	86.67
67 [83]	ginsim.org/node/71	56	50	50	89.29	89.29
68 [84]	ginsim.org/model/SP	19	17	17	89.47	89.47
69 [81]	ginsim.org/node/37	10	9	9	90.0	90.0
70 [81]	ginsim.org/node/37	10	9	9	90.0	90.0
71 [85]	ginsim.org/model/signalling-prostate-cancer	133	121	121	90.98	90.98
72 [86]	ginsim.org/node/229	133	124	122	93.23	91.73
73 [87]	ginsim.org/model/T2DM	26	24	24	92.31	92.31
74 [88]	ginsim.org/node/194	14	13	13	92.86	92.86
75 [86]	ginsim.org/node/229	53	50	50	94.34	94.34
76 [89]	ginsim.org/node/240	18	17	17	94.44	94.44
77 [90]	ginsim.org/node/191	20	19	19	95.0	95.0
78 [91]	ginsim.org/node/234	61	58	58	95.08	95.08
79 [90]	ginsim.org/node/191	32	31	31	96.88	96.88
80 [92]	ginsim.org/node/216	34	33	33	97.06	97.06
81 [80]	ginsim.org/node/82	7	7	7	100.0	100.0
82 [93]	ginsim.org/node/189	14	14	14	100.0	100.0
83 [7]	ginsim.org/node/4	10	10	10	100.0	100.0
84 [93]	ginsim.org/node/189	13	13	13	100.0	100.0
85 [80]	ginsim.org/node/82	7	7	7	100.0	100.0
86 [94]	ginsim.org/model/p53-Mdm2	6	6	6	100.0	100.0

Table S3: Large-scale application of BBE on the repositories GINsim and Biomodels. *Model ID* gives the model identifier as in Fig. 2 and a reference. *URL* allows to download the model from the repositories. *Size* presents the number of variables in the original BN, and in its IS and maximal reduction (N, N_i, N_m resp.). The last column contains the ratios: $R_i = N_i/N$ and $R_m = N_m/N$. In most of the cases, BBE took less than a second. The largest runtime is about 2 seconds.

Model ID	Original		IS Reduced		Max Reduced	
	N	Time (s)	N_i	Time (s)	N_m	Time (s)
1 [50]	26	time-out	7	0.046	4	0.048
2 [51]	19	21.739	11	0.433	3	0.037
3 [50]	24	time-out	11	5.768	4	0.041
4 [52]	57	time-out	34	time-out	11	0.453
6 [53]	18	12.470	13	2.749	4	0.038
8 [50]	16	2.581	8	0.265	5	0.229
9 [50]	23	339.396	13	0.259	8	0.042
10 [50]	19	20.333	8	6.931	7	5.472
11 [50]	24	time-out	11	0.089	9	0.055
12 [55]	37	time-out	16	2.492	14	0.954
13 [50]	34	time-out	23	417.864	15	1.191
14 [50]	18	7.836	11	4.178	8	2.759
15 [56]	30	time-out	25	time-out	14	0.485
16 [54]	24	time-out	15	6.602	12	0.179
21 [50]	18	11.608	11	0.116	10	0.079
22 [61]	36	time-out	23	440.921	21	96.438
30 [55]	19	21.901	14	0.568	12	0.180
35 [67]	28	time-out	22	167.489	20	39.467
40 [45]	16	3.554	12	0.265	12	0.251
41 [69]	20	37.951	15	0.972	15	0.968
43 [45]	17	9.598	13	1.751	13	1.256
44 [70]	30	time-out	23	346.175	23	338.690
46 [72]	28	time-out	22	160.363	22	166.330
53 [66]	16	3.800	13	0.647	13	0.652
57 [45]	18	12.099	15	1.270	15	1.307
59 [79]	26	time-out	23	425.386	22	202.055
62 [81]	14	0.655	12	0.155	12	0.185
63 [82]	21	88.812	18	10.205	18	10.148
65 [75]	15	1.159	13	0.326	13	0.305
68 [84]	19	21.972	17	4.465	17	4.397
74 [88]	14	2.228	13	0.894	13	0.897
76 [89]	18	11.451	17	4.376	17	4.453
77 [90]	20	43.676	19	23.144	19	22.595

Table S4: The table displays the results of the large-scale validation for STG generation. The first column contains the model identifier. Then, each 2-columns block *Original*, *IS Reduced*, and *Max Reduced* contains information on STG generation on the original BN and its IS and maximal reductions, respectively. In particular, N, N_i and N_m contain the number of variables, while *Time* contains the time in seconds, averaged over 3 runs, for STG generation by PyBoolNet.

Model ID	Original			IS Reduced			Max Reduced		
	N	Attractors	Time (s)	N_i	Attractors	Time (s)	N_m	Attractors	Time (s)
1 [50]	26	16384	16.771	7	2	0.002	4	2	0.002
2 [51]	19	2832	2.296	11	16	0.011	3	2	0.002
3 [50]	24	64	0.067	11	2	0.002	4	2	0.002
4 [52]	57	9984	20.124	34	4	0.009	11	2	0.002
5 [53]	10	125	0.072	2	2	0.001	2	2	0.001
6 [53]	18	339	0.283	13	14	0.011	4	2	0.002
7 [54]	8	24	0.030	4	3	0.002	2	2	0.001
8 [50]	16	512	0.367	8	2	0.0026	5	2	0.002
9 [50]	23	512	0.473	13	2	0.003	8	2	0.002
10 [50]	19	4110	3.284	8	2	0.002	7	2	0.002
11 [50]	24	8192	7.866	11	2	0.002	9	2	0.002
12 [55]	37	15459	21.847	16	16	0.014	14	12	0.010
13 [50]	34	1536	1.952	23	3	0.005	15	2	0.003
14 [50]	18	256	0.203	11	2	0.002	8	2	0.002
15 [56]	30	9	0.019	25	3	0.005	14	2	0.003
16 [54]	24	1596	1.572	15	9	0.009	12	4	0.004
17 [57]	92	7360	24.031	72	50	0.144	46	2	0.008
18 [58]	103	–	time-out	54	9	0.145	52	9	0.126
19 [59]	66	77876	235.756	43	2	0.008	35	2	0.005
20 [60]	45	1	0.009	24	1	0.002	24	1	0.003
21 [50]	18	384	0.313	11	3	0.003	10	2	0.002
22 [61]	36	86358	118.792	23	12	0.014	21	12	0.013
23 [61]	71	121976	359.252	44	12	0.025	42	12	0.023
24 [62]	96	4	0.030	81	2	0.015	57	2	0.009
25 [63]	82	1	0.013	49	1	0.005	49	1	0.005
26 [3]	10	10	0.011	7	3	0.002	6	2	0.002
27 [3]	10	10	0.011	7	3	0.002	6	2	0.002
28 [64]	41	990	1.50	27	6	0.010	25	5	0.008
29 [65]	218	–	time-out	140	68	2.433	136	68	2.182
30 [55]	19	308	0.255	14	10	0.009	12	7	0.006
31 [3]	9	7	0.009	7	4	0.003	6	3	0.002
32 [3]	9	7	0.009	7	4	0.003	6	3	0.002
34 [30]	42	2	0.010	37	2	0.005	29	2	0.004
35 [67]	28	97	0.121	22	23	0.025	20	23	0.023
36 [31]	40	8	0.019	29	2	0.005	29	2	0.005
37 [68]	48	22	0.047	38	4	0.009	35	3	0.007
38 [45]	53	40	0.138	41	17	0.038	39	17	0.034
39 [30]	128	8	0.072	103	2	0.0232	95	2	0.021
40 [45]	16	20	0.023	12	5	0.005	12	5	0.004
41 [69]	20	1	0.007	15	1	0.002	15	1	0.002
42 [67]	33	71	0.104	26	29	0.038	25	29	0.035
43 [45]	17	22	0.027	13	4	0.004	13	4	0.004
44 [70]	30	6	0.026	23	6	0.008	23	6	0.008
45 [71]	56	1972	4.250	44	2	0.009	43	2	0.009
46 [72]	28	2	0.009	22	2	0.003	22	2	0.004
47 [66]	39	3	1.387	31	3	0.073	31	3	0.072
48 [73]	35	62	0.102	31	8	0.015	28	8	0.014
49 [74]	10	1	0.005	8	1	0.002	8	1	0.002
50 [75]	10	48	0.032	8	12	0.007	8	12	0.007
51 [76]	31	53	0.072	26	23	0.028	25	17	0.021
52 [77]	37	57	0.136	32	2	0.008	30	2	0.007
53 [66]	16	2	0.008	13	2	0.003	13	2	0.003
54 [50]	11	4	0.007	9	2	0.002	9	2	0.002
55 [66]	50	1	2.343	41	1	0.134	41	1	0.134
56 [30]	110	4	0.030	91	2	0.016	91	2	0.016
57 [45]	18	22	0.027	15	5	0.005	15	5	0.006
59 [79]	26	6	0.017	23	3	0.006	22	3	0.006
60 [80]	7	1	0.005	6	1	0.002	6	1	0.001
61 [80]	7	1	0.005	6	1	0.001	6	1	0.001
62 [81]	14	1	0.006	12	1	0.002	12	1	0.002
63 [82]	21	8	0.014	18	8	0.009	18	8	0.009
64 [76]	29	27	0.096	27	15	0.021	25	8	0.013
65 [75]	15	48	0.041	13	12	0.010	13	12	0.010
66 [20]	60	264	0.676	52	6	0.022	52	6	0.021
67 [83]	56	3	0.034	50	3	0.016	50	3	0.019
68 [84]	19	19	0.024	17	5	0.006	17	5	0.006
69 [81]	10	13	0.014	9	5	0.004	9	5	0.004
70 [81]	10	2	0.006	9	2	0.002	9	2	0.002
71 [85]	133	7008	108.475	121	4	0.103	121	4	0.102
72 [86]	133	11	2.882	124	5	2.118	122	5	2.161
73 [87]	26	32	0.045	24	8	0.012	24	8	0.012
74 [88]	14	4	0.009	13	2	0.003	13	2	0.003
75 [86]	53	10	0.502	50	5	0.220	50	5	0.217
76 [89]	18	5	0.012	17	5	0.006	17	5	0.006
77 [90]	20	13	0.020	19	7	0.008	19	7	0.008
78 [91]	61	32	0.115	58	7	0.034	58	7	0.033
79 [90]	32	15	0.032	31	9	0.018	31	9	0.018
80 [92]	34	20	0.048	33	10	0.031	33	10	0.030

Table S5: The table contains the large-scale validation for the attractor analysis. The first column is the model identifier and its reference. Each 3-columns block *Original*, *IS Reduced*, and *Max Reduced* contains information on attractor computation on the original BN and its IS and maximal reductions, respectively. In particular, N, N_i, and N_m contain the number of variables, while *Attractors* contains the number of attractors computed by tool BNS, and *Time* the time in seconds to obtain them averaged over 3 runs.
